# Advances in Molecular Function and Recombinant Expression of Human Collagen

**DOI:** 10.3390/ph18030430

**Published:** 2025-03-18

**Authors:** Wenli Sun, Mohamad Hesam Shahrajabian, Kun Ma, Shubin Wang

**Affiliations:** 1 National Key Laboratory of Agricultural Microbiology, Biotechnology Research Institute, Chinese Academy of Agricultural Sciences, Beijing 100086, China; hesamshahrajabian@gmail.com; 2 Hantide Biomedical Group Co., Ltd., Zibo 256300, China; makun_88@163.com

**Keywords:** biological properties, collagen structure, extracellular matrix, molecular function, recombinant collagen

## Abstract

Collagen is the main protein found in skin, bone, cartilage, ligaments, tendons and connective tissue, and it can exhibit properties ranging from compliant to rigid or form gradients between these states. The collagen family comprises 28 members, each containing at least one triple-helical domain. These proteins play critical roles in maintaining mechanical characteristics, tissue organization, and structural integrity. Collagens regulate cellular processes such as proliferation, migration, and differentiation through interactions with cell surface receptors. Fibrillar collagens, the most abundant extracellular matrix (ECM) proteins, provide organs and tissues with structural stability and connectivity. In the mammalian myocardial interstitium, types I and III collagens are predominant: collagen I is found in organs, tendons, and bones; collagen II is found in cartilage; collagen III is found in reticular fibers; collagen IV is found in basement membranes; and collagen V is found in nails and hair. Recombinant human collagens, particularly in sponge-like porous formats combined with bone morphogenetic proteins, serve as effective scaffolds for bone repair. Due to their biocompatibility and low immunogenicity, collagens are pivotal in tissue engineering applications for skin, bone, and wound regeneration. Recombinant technology enables the production of triple-helical collagens with amino acid sequences identical to human tissue-derived collagens. This review summarizes recent advances in the molecular functions and recombinant expression of human collagens, with a focus on their biomedical applications.

## 1. Introduction

Collagens derived from animal tissues pose risks of allergic reactions, immunogenicity, and pathogen transmission [1]. In contrast, recombinant human collagens offer advantages such as high purity, non-animal sourcing, and elimination of viral or prion contamination risks [2,3,4]. Skin defects caused by trauma or pathology often lead to infections, inflammation, chronic wounds, and scarring [5,6]. As a major ECM component in skin, collagen is essential for maintaining structural integrity and physiological function [7]. Fibroblasts and collagen collectively regulate dermal physiology, including wound healing and tissue remodeling [7,8,9,10]. Patrawalla et al. [11] demonstrated that collagen interacts with glycoproteins, glycosaminoglycans, and proteoglycans to form a complex ECM, which underpins tissue diversity and mechanical resilience.

Collagen’s low antigenicity, biocompatibility, and ability to promote cell attachment and migration make it a cornerstone of wound-healing biomaterials [10,11]. Additionally, collagen interacts with host-defense proteins (e.g., C1q collectins and macrophage scavenger receptors) and forms a network of interconnected fiber bundles. These bundles, composed of fibrous protein assemblies, provide skin with tensile strength, elasticity, and mechanical stability [12].

Humans produce at least 28 collagen types, categorized into fibril-forming collagens (e.g., types I, II, III, V, and XI) and nonfibrillar collagens [13,14]. Collagen I, the primary ECM component in human skin, is widely used in dermatological and cosmetic applications due to its role in maintaining tissue biomechanics [15,16,17].

Recombinant human collagen was firstly modified with methacrylate anhydride (MA) to synthesize the derived collagen hydrogels RHCMA, and MI-RHCMA hydrogel could induce the regeneration of damaged corneal stroma in vivo [18]. Collagen fibril length and angle do not significantly change with region, age, or vitreoretinal adhesive strength, and collagen fibrils in the vitreous cortex have been proved to penetrate the inner limiting membrane of the retina at the vitreous base [19,20,21]. The preparation process of the novel recombinant human collagen hydrogel (RHCH) is low-cost and simple, as RHCH can be effective in migration of cells essential for wound healing, as well as positively influencing this process in in vitro trials [22]. A combination of different bioceramics and collagen has been found to be more effective in enhancing the amount of bone formation, as collagen-based scaffolds usually present from low osteoconductive capacity in comparison with bioceramic materials [21,22]. The goal of this manuscript is to survey and study the recent findings in recombinant expression and molecular function of human collagen. This review also discusses biosynthesis, structure, isolation techniques, sources, and characterization of the physicochemical properties of different collagens. Important information on various collagens of animal by-products and extraction methods of collagens are shown in Table 1. The collagen protein family and associated disorders are shown in Table 2.

## 2. Classification and Function of Human Collagen

Collagens present a common structural basis known as the abundance of repeating Gly-C-Y amino acid triplets, where Y and X are usually represented by 4-Hyp and Pro, respectively [23,24,25]. In some researches, it is noted that collagen from human bone has a specific amino acid sequence from which it is possible to assess the C/N ratio, as the C/N ratio is applied to evaluate availability of collagen derived from bone for isotope measurement [26]. It is also discovered that collagen and human amniotic membrane (hAM) are usually applied biomaterials in regenerative tissue repair, such as peripheral nerve regeneration, and hAM nerve wraps are an important biomaterial which is effectual for enhancing results of peripheral nerve regeneration. Moreover, compared to collagen nerve wraps, hAM nerve wraps may lead to better functional recovery and superior nerve regeneration [27]. Rosu et al. [28] found that three-dimensional biopolymers with collagen are important models for extracellular matrix researches, and human apolipoprotein A-I (apoA-I) bound less to matrices with heparin and collagen than to those with only collagen. Human-like collagen (HLC) is bio-safe, biomimetic, and hydrophilic, and HLC self-assembles loaded on the oxidized-modified hyaluronic acid (oxi-HA) showed higher biological activity characteristics than hyaluronic acid, which proves its importance as a new component of multifunctional HA-based wound-healing generation [29]. Voziyan et al. [30] mentioned that the advanced glycation end products (AGEs) play an important function in pathogenesis of diabetic complications, and the map may improve evaluation of pathogenic mechanisms involving collagen I and AGEs. The high amount of secondary amino acids and Gly in these triplets can induce stabilization of the 3_10_-helical conformation of individual collagen chains and promote the assembly of triple helices [31].

Collagen peptides are effective in decreasing excessive hair shedding and thinning associated with patterned hair loss or aging [32]. Compared to tubes made of collagen hydrogels, collagen filaments have increased mechanical characteristics, and endothelial cells (ECs) cultured within the hollow fibers under perfused conditions indicate obvious cell−cell contacts, high cell viability, and remodeling of the underlying matrix [33]. The lipid extraction may not be a necessary stage of bone collagen isolation, especially for non-marine diet populations. Although the collagen-based scaffold plays the appropriate biological roles in tissue engineering, there are still some limitations because of its rapid degradation rate and poor mechanical strength [34,35,36]. 

Recombinant human collagen (RHC) has high resistance to gastrointestinal digestion, and RHC digestates can reduce reactive oxygen species (ROS) levels by increasing antioxidant enzyme activities, improve the synthesis and inhibition the degradation of ECM and have higher protective impact on fibroblasts than animal collagens [37]. The mechanical behavior of human periodontal ligament (PDL) follows the trend of hyperelastic materials and noticeably relies on the spatial angles of internal collagen fibers. This model could significantly show the hyperelastic characteristics of human PDL [38]. On the basis of electron microscopic analyses and fibrillation kinetics, it is reported that lutein does not interfere with the fibrillation procedure of collagen. However, it increases the lateral fusion of collagen fibrils, leading to the formation of compact bundles of thick fibrils under physiological conditions [39]. This demonstrates the interaction between carotenoid and collagen, indicating the unique property of lutein in bundling collagen fibrils and its potential in application in tissue engineering [39]. Ben et al. [40] described that the recombinant human collagen gel is considered to be an appropriate alternative for the heterologous/homologous skin disorders. Type VII collagen (COL7) can be encoded by COL7A1, and its mutations may cause recessive dystrophic epidermolysis bullosa (RDEB) that is a severe skin fragility disease. COL7 can reduce anchoring, repair defects and enhance skin integrity in RDEB patients [41]. Fischer et al. [42] concluded that the collagen fiber arrangement is important for constitutive description of anisotropic mechanical reaction of the arterial wall. Guo et al. [43] designed a novel recombinant human collagen (rhCol), and on the basis of RT-qPCR and immunofluorescence staining, it was proved that rhCol/basic fibroblast growth factor (bFGF) can promote expression of bone-related proteins, which shows its importance as a suitable scaffold in clinic applications.

Studies showed that collagen hydrogel has important biocompatibility and considerable capacity to increase expression and cell viability of stemness-associated genes, including SRY-box transcription factor 2 (SOX2), Nanog homeobox (Nanog), and organic cation/carnitine transporter4 (Oct-4) of human umbilical cord mesenchymal stem cells (uMSCs) [44]. The primary human intestinal myofibroblast (HIMF)-derived ECM in inflammatory bowel disease (IBD) binds a remarkably increased number of T cells which is dependent on integrin αvβ1 and collagen VI, and the expression of Collagen VI is a risk parameter for the future complicated Crohn’s disease (CD) course [45]. Members of the microRNA-29 (miR-29) gene family could inhibit proliferation and induce apoptosis in human fetal scleral fibroblasts (HFSFs), with important role in scleral remodeling, and it could also downregulate heat shock protein 47 (Hsp47), COL1A1 and Smad3 in HFSFs [46]. Kaviani et al. [47] found that the reconstruction of the destructed matrix with collagen microspheres and alginate hydrogels can be useful to increase the culture of the islets through TUNEL positive cells and the reduction of activated caspase-3. It is discovered that the newly synthesized collagen/poly(3-hydroxybutyrate) (COL/P3HB) scaffold increased the colon anastomosis healing [48]. Collagen types V and I fibrils have important functions in the structural backbone of bone [49,50], collagen types XI and II usually contribute to the fibrillar matrix of articular cartilage [49], and collagen types XIV, XII, and IX have an important function in regulating the diameter of collagen fibrils [51]. In collagen types X, VIII, VII, VI, and IV, non-collagenous domains have significant functions in network formation and aggregation [52], types XI and V collagens are formed as heterotrimers of three different α-chains (α1,α2,α3), and types XIV and XII collagens are structurally associated with short-chain collagens [52].

Fibrillar collagens are collagen I which can be found in bone, skin, cornea, tendon, vascular ligature, and organs; collagen II which can be observed in vitreous body, cartilage, and gristle; collagen III that can be observed in reticulate, commonly found together with collagen type I in skin, vessels, intestine, and uterus; collagen V in skin, cell surfaces, cornea, placenta, skin, and hair; collagen XI in intervertebral disc and gristle; collagen XIV which can be found in gristle, skin, tendons, bones, eye, nerves, and vessels; and collagen XXVII in gristle.

**Figure 1:**
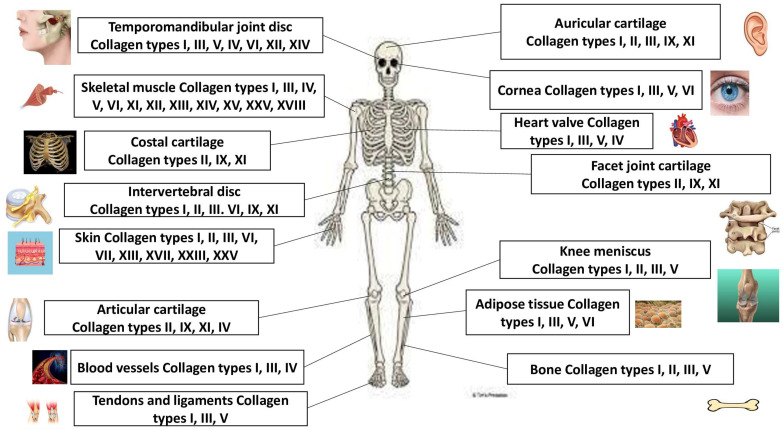
The collagen protein family and their main locations.

Nonfibrillar collagens are the collagens as follows. Collagen IV can form basal lamina, the epithelium-secreted layer of the basement membrane, and capillaries. Collagen VII can be found in umbilical cord, amniotic fluid, skin, bladder, and mucous membranes. Collagen VI is in cornea, gristle, vessels, bones, and skin. Collagen XXVIII is in nervous system cells, and collagen XXIX can be observed in skin. Collagen VIII can be found in gristle, vessels, bones, brain, kidneys, skin, and heart; collagen V can be observed in gristle; collagen XIII can be found in skin, endothelial cells, heart, eye, and skeletal muscles; collagen XVII can be observed in skin; collagen XXIII can be observed in metastatic carcinogenic cells. Collagen XXV can be discovered in testicles, brain, heart and eye; collagen IX can be found in the gristle, vitreous body, and cornea; collagen XII can be discovered in the tendons, skin, and gristle. Collagen XVI is in the kidneys, muscle, heart, and skin; collagen XIV is in gristle, skin, tendons, bones, eye, nerves, and vessels; collagen XIX can be found in the prostate gland, placenta, spleen, kidneys, liver, and skin; collagen XX is in corneal epithelium; collagen XXI is in vessels, skeletal muscles, placenta, kidneys, heart, and stomach. Collagen XXII can be found in tissue connections; collagen XXVI is in ovaries and testicles; collagen XXIV is in cornea and bones; collagen XV is in placenta, kidneys, testicles, skin, ovaries, heart, and capillary vessels; and collagen XVIII can be found in lungs, liver, and kidneys. The collagen protein family and their locations are presented in Figure 1.

## 3. Collagen I

The most abundant protein in the body, especially in the connective tissues, is type I collagen [53], and it is the main component of the extracellular matrix [53], which is found in tissues tendon, bone, and skin [54]. It forms a heterotrimer consisting of two α1(I) chains and a genetically distinct α2(I) chain. The α111(I) homotrimer has been also found in different limited situations in vivo such as cancer tissues, fibrotic, osteoarthritis, and fetal [55]. It is considered as the gold standard biomaterial for the manufacturing of medical devices used in healthcare [55], and collagen type I from equine tendon preserves its native fibrillar conformation [54,55]. Taga et al. [56] found that the most suitable method which can clearly detect the rare form of type I collagen is LC-MS method.

The most abundant collagen in long-lived extracellular matrix proteins is type I collagen, as they can accumulate higher levels of advanced glycation end products (AGEs) and glycoxidation of collagen I. Glycation, together with other proteins of bone ECM, has been proved to contribute to the diabetes-related increase in bone fragility [57]. Voziyan et al. [57] showed levels and sequence positions of main AGEs in human bone collagen I, and molecular modeling revealed the effects of AGEs on collagen structure in diabetic bone. Wu et al. [58] reported the introduced recombinant human collagen I in fabricating a new hydrogel loaded with human umbilical cord mesenchymal stem cell (hUCMSC)-derived exosomes, which can significantly promote skin regeneration and wound closure, showing high potential in severe skin wound healing treatment. Collagen I can inhibit the accumulation of damaged mitochondria in ultraviolet B (UVB)-treated cells, and precoating culture plates with collagen I can reduce UVB-induced HaCaT cell apoptosis [59,60,61,62,63]. Hu et al. [61] reported that the transplantation of the menstrual blood mesenchymal stem cell (MBMSC)-loaded collagen scaffold (CS) reduced collagen I and enhanced the CK 18 significantly, and according to the findings of immunoblotting results, the upregulation of CK 18 and downregulation of collagen I were proved. Marin et al. [64] found that treatment of older and young adult human skin cells with sacubitril and valsartan may induce a five-fold boost in collagen type I production in the young cells and a four-fold increase in collagen type I in older adult cells.

It is noted that the length and density of collagen I fibers in triple-negative breast cancer make an important contribution to patient prognosis and tumor size, and it may allow breast cancer cells to metastasize to the lungs [65,66]. Its matrices have been applied both as three-dimensional environments and tissue engineering for biological research [67,68,69]. Stromal collagen type I may predict chemotherapy response, and protein expression and low stromal collagen type I mRNA are associated with unfavorable tumor characteristics in breast cancer [70,71]. Zhang et al. [72] reported that collagen type I has a relatively stable quality while completely resistant to digestion due to its complex triple helix structure. It inhibits autophagy by upregulating the YAP-mTOR pathway, and it can improve lipid metabolism and glucose through the YAP-autophagy axis [73,74,75]. Collagen type I proteolysis by matrix metalloproteinase (MMP)-2 activates focal adhesion kinase (FAK) in aortas and induces vascular smooth muscle cell (VSMC) proliferation and hypertrophic remodeling [75]. Collagen type I produced by pancreatic cancer cells is the oncogenic homotrimer variant, and it may enrich T cells and promote a beneficial tumor microbiome [74,75]. Preston et al. [75] also reported that collagen type I plays an important role in treatment of breast cancer. It may present challenges as a bioink [76]. Tumor-associated collagen I contributes to aberrant transcriptional activity. This promotes intracellular signaling that induces mechanotransduction-associated procedures which may give rise to chemoresistance [77].

## 4. Collagen II

Collagen type II is a main structural protein of cartilaginous tissues [78], which is first synthesized as procollagen [79,80,81,82]. As one of the basic components of the cartilage matrix, it can improve bone health and promote chondrocyte differentiation, especially for rheumatoid arthritis [83,84]. Collagen II type is heterotypically associated with type XI and type IX collagens [85]. It is the main constituent of cartilage tissue, and its devices in cartilage engineering are linked with suboptimal functional therapeutic outcomes [86]. It is usually isolated from the cartilages of terrestrial animals such as bovine and swine [87]. As collagen type II is a fibrillar collagen, it includes 95% of the collagen and nearly 60% of the dry weight of cartilage [88,89]. In fact, collagen is a polymer which exists as a triple helix with chains connected by hydrogen bonds [90], and types II and I collagens are different in amino acid [91,92]. Type I collagen is more effective in increasing surimi gel properties than type II [93], and the expression of collagen type II is regulated by the Sox trio of transcription factors [94,95]. Its strength and stability can provide the tissue with resiliency and integrity to stress [96,97], and the amount of collagen type II is higher in the tissue around the calcium deposit [98,99]. During skeletal development, type II collagen plays an important role in the embryonic cartilaginous skeleton [100,101]. Type II collagen could be induced to strengthen a weakened cornea [101], and degradation of type II collagen caused by pro-inflammatory cytokines is one of the main pathological traits of osteoarthritis (OA) [102,103]. A type II collagen-specific neoepitope is the first technically robust serological biomarker assay related to osteoarthritis cohorts, which proves its role as a translational biomarker for cartilage degradation [104,105]. The metabolic disorder and degradation of collagen type II in human cartilage can influence the development of osteoporosis and osteoarthritis [106].

## 5. Collagen III

Collagen III can be found in the endometrium and consists of a main portion of the extracellular matrix [107]. It possesses the ability to influence cell proliferation, adhesion and migration and bind to integrins [107]. As the second most abundant collagen, type III collagen is extensively distributed in various connective tissues, such as the vascular system and internal organs [108]. It is found that both collagen III and collagen I play significant roles in fibroblast activation [108]. It is reported that it plays important roles in normal cardiovascular development in humans and wound healing [109]. Both types I and III collagens are main ingredients in tissue regeneration and wound healing [110,111]. Recombinant human collagen III is synthesized via a re-optimized gene sequence design according to the primary functional domains and the features of human collagen [112]. Type III and type I collagens bind to collagen receptors, perform various biological roles, such as a supportive function in cell migration, differentiation, and adhesion as well as M2 macrophage polarization, and promote ECM, platelet aggregation, and angiogenesis [111,112]. Li et al. [112] found that recombinant human collagen (rhCOL)-derived material with high cell adhesion characteristic can promote migration, HULF adhesion, and ECM synthesis, regulate the metabolism of human uterosacral ligament fibroblast (HULFs), influence the focal adhesion kinase (FAK)/RhoA/ROCK signaling pathway, improve pelvic floor function in pelvic floor dysfunction rats and reduce remodeling effects. The collagen α1(III) chain (COL3A1) is considered as an important structural protein on the surface of human skin [113]. Human collagen type III (hCOL3A1) associates to the fibril-forming collagens, which is distributed in an extensive connective tissue-like vascular system with important roles in collagen fibrillogenesis and wound healing [114]. It is reported that a human type III collagen (hCOLIII)-based ECM-mimetic coating can be used as a tailored blood-contacting component for cardiovascular stents, as it suppressed inflammatory response, increased endothelialization and inhibited the excessive neointimal hyperplasia [114,115,116]. As one of the main structural proteins, collagen III is classified as one of the main fibrillar collagens [116], and it can influence the occurrence of incisional hernia and play a considerable role in maintaining tissue tensility and elasticity [117,118]. Both types I and III collagens are the major components of the interstitial matrix [119,120]. It plays a notable role in different inflammation-associated pathologies such as arthropathies, lung injury, kidney fibrosis, liver disease, vascular disorders, and hernia [121,122]. Collagen type III promotes migration and cell viability of endometrial stromal cells [123,124]. Both collagen types III and I can reduce aged skin [125], and their accumulation can be found in fibrotic diseases. They can also regulate type I collagen fibril formation [126,127,128]. Type III collagen is less abundant in women with diastasis recti than in those without the condition [129,130], and it can influence the aggrecan network compression possibly via mediating collagen network integration and the aggrecan [131]. It can serve as a potential treatment method for breast cancer patients to improve prognosis of patients [132] and reduce deposition in metastatic prostate cancer [133]. Collagen type III promotes the proliferation of myoblasts, and it has the capability to inhibit the differentiation of myoblasts [134,135]. Collagen type III can be considered as an anti-osteoarthritic candidate [136], and it can regulate myofibroblast differentiation and scar formation after cutaneous injury [137,138].

## 6. Collagen V

Collagen V is a fibrillar collagen which is needed for the normal formation of collagen III and I fibrils by determining and initiating their properties and diameters [139]. It can contribute to the corneal stroma, bone matrix, and the interstitial matrix of liver, muscles, placenta, and lungs [140]. Elhers−Danlos syndromes (EDS) are the main cause of collagen V mutations [140], and collagen V can regulate collagen fiber assembly and geometry and link stromal and strength of collagen to the basement membrane [141]. The most common collagen V isoform is α1(V)_2_ α2(V) found in cornea, but other isoforms include the [α1(V)α2(V)α3(V)] form, an α1(V)_3_ homotrimer, and hybrid type V/XI forms [142]. The deficiency of collagen V can enhance scar size after acute heart injury [143,144,145], and increases in collagen V are associated with an increased number of TGF-β1, fibroblasts. These changes are more pronounced in chronic, long-standing persistent atrial fibrillation (cAF) patients than those in paroxysmal AF (pAF) patients, which proves that AF is significantly associated with increased expression levels of TGF-β1 and collagen V, showing its important function in the pathogenesis of atrial fibrosis [146]. Collagen V most probably acts as fibril diameter regulators as they can keep a bulky amino-terminal portion linked to the triple helix after their final extracellular processing [145,146]. It has a significant function in connective tissue contraction, especially during tissue regeneration and wound healing [146]. Reduction of collagen type V can decrease aberrant cell clustering and cell density in both hyaline and fibrous layers, and its loss may lead to reduced *β*-catenin expression and cell proliferation in the fibrous layer [147]. Collagen V plays an important role in the formation of human islet organoids which harbor all main pancreatic endocrine cell types, and it can augment the generation of endocrine which secretes glucagon and glucose level-regulated insulin [148]. It can induce a specific transcriptional signature in murine splenic B cells as well as rapid production of anti-collagen V antibodies [148]. Collagen type V turnover may be significantly important in the pathogenesis of ankylosing spondylitis [147,148]. It can influence migration, adhesion, viability, and metastatic potential of pancreatic cancer cells, and its downstream pathway exerts possible therapeutic targets in pancreatic ductal adenocarcinoma (PDAC) [149]. It also has a significant function in modifying cell behavior during remodeling and development when very soft tissues are present [150]. Pre-treatment by intravenous injection of collagen V may inhibit bleomycin-induced pulmonary fibrosis by suppressing IL-6 and IL-17 production [151].

## 7. Collagen IV

Nonfibrillar collagens arise from disruptions in the Gly-X-Y repeats of the alpha chains [152,153,154,155,156,157]. Instead of forming fibrils, these nonfibrillar collagens form mesh-like networks in the ECM, such as collagen IV found in basement membranes [158,159,160,161,162,163,164,165,166,167,168,169,170,171,172]. They are expressed in smaller amounts [173,174,175,176,177,178,179,180] and influence the shape and fiber thickness of type I collagen or anchor the groups of fibers to each other and surrounding tissues [181,182,183,184,185,186,187,188,189,190]. They are rich in proline, glycine, and hydroxyproline and form triple-helical units [191,192,193,194,195,196,197,198,199,200,201,202,203,204,205,206,207,208,209,210]; however, the helical region is short or interrupted [211,212,213,214,215,216,217,218,219,220,221,222,223,224,225]. The nonfibrillar collagens form basement membranes, anchors, and microfibrils [226,227,228,229,230,231,232,233,234,235,236,237,238,239,240]. The N- and C-terminal regions of the triple helices are not cleaved off, as in fibrous collagens, nor are they cross-linked by oxidized lysine residues [241,242,243,244,245,246,247,248,249,250,251,252,253,254,255,256,257,258]. The main cross-links found in types IV, VII, and XVII collagens are mediated by cysteine disulfide bonding, mostly within their C-terminal regions [259,260,261,262,263,264,265,266].

Collagen IV is an important constituent of the glomerular basement membrane (GBM), and its synthesis starts with the transcription of genes encoding the alpha chains into messenger RNA within the cell nucleus [152]. It can be found in several tissues such as vascular endothelial and skeletal muscles [153]; moreover, the main ingredient of the basement membrane in epithelial tissues is collagen IV [154]. Its two important functions are to enhance development of architecture tissues and structural stability [155]. In fact, it includes three α-chains of primarily repeating Gly-Xaa-Yaa triplets, which induces each α-chain to adopt a left-handed poly-pro-II helix [154,155,156,157]. As the major collagen ingredient of the basement membrane [173] and a heterotrimeric molecule, it has the globular C-terminal NC1 domain, a central triple-helical domain, and the N-terminal 7S [174]. It is a network-forming collagen which underlies endothelial and epithelial cells and acts as a barrier between tissue components [175]. It has six highly homologous polypeptide chains, namely α1(IV) to α6(IV) with each including the carboxy-terminal, triple-helical, and amino-terminal domains [176]. Transforming growth factor (TGF) beta can regulate type IV collagen production in stromal corneal fibroblasts [177]. It is also reported that collagen IV is the main backbone of the basement membrane [178,179]. It has capability to downregulate ERK which may induce Mucin 5AC (MUC5AC) secretion [180], and it can be useful in treatment of breast cancer [179,180]. It is an appropriate indicator of basement membrane damage in workers occupationally exposed to volatile organic compounds [181], and it is reported as an important agent for treatment of non-small cell lung cancer cells [182,183]. It has also an important function for treatment of tumor-initiating cells in oral cancer [184,185]. Collagen IV could shape organs and cells by exerting a mechanical constricting tension [185]. It can influence the invasive behavior of trophoblast cells at the implantation site and also represents a structural protein providing tissue integrity [186]. It has a dynamic contractile activity in reaction to electrical pulse stimulation which proves its role in the design of myoblast-based therapies [187]. Collagen IV could predict the presence of advanced fibrosis in non-alcoholic fatty liver disease (NAFLD) patients [188], and it has also shown effective function relevance to ocular diseases [189].

## 8. Collagen VI

Collagen VI is an important component of the extracellular matrix in all connective tissues [190], which can be found at the interface between the interstitial matrix and basement membrane [191]. It is both a signaling and a structural protein [191], which can be found in tendon, bone, cartilage, cornea, and muscles [192]. The major collagen type VI genes are *COL6A3*, *COL6A2*, and *COL6A1*, which are expressed by interstitial muscle [193]. These genes are primarily found in close connection with basement membranes with important functions in progenitor cell attachment and matrix conformation [192,193]. The incorporation of collagen type VI in immunoisolated human islets supports survival of human pancreatic islets and in vitro viability [194], and collagen VI in hippocampal slices could decrease glutamate release, which leads to a decline in neuronal excitability [195]. Collagen VI deposition is associated with higher T cell density in prostate cancer patients, and it can directly influence lung epithelial cell phenotype in vitro [196]. It is also reported that it has an important function in the preterm chronic lung diseases, including Bronchopulmonary Dysplasia (BPD) [197].

## 9. Collagen VII

As the major component of anchoring fibrils, collagen type VII is important for skin integrity [156,157], and it is also vital for linking various skin layers together [158]. It includes a central collagenous triple-helical domain flanked by two non-collagenous domains, namely NC2 and NC1 [157,158]. Functional defects in type VII collagen are accountable for recessive dystrophic epidermolysis bullosa (RDEB) and severe autosomal recessive kinds of the skin blistering disease [158]. It is important for stability and function of the ECM as it is an anchoring fibril collagen [198], and it is basically synthesized by fibroblasts and keratinocytes [199]. It is involved in systemic sclerosis, chronic obstructive pulmonary disease, and Crohn’s disease [189,199]. Type VII collagen has a positive effect on treatment of brittle bone disease known as osteogenesis imperfecta (OI), and the initial synthesize of collagen VII is via basal keratinocytes and dermal fibroblasts [199,200]. It is originally known because of its unusually lone and an extended molecule [199,200]. The lack of type VII collagen disrupts cellular proteostasis and acts as a scaffold to load Transport and Golgi Organization-1 (TANGO1)-mediated COPII carriers by binding both the coat protein II complex (COPII) and TANGO1 cargo [201]. It is important for the stability of the skin and other epithelial organs [200,201], and its genetic loss may cause dystrophic epidermolysis bullosa, which can manifest with fibrosis and chronic skin fragility [202]. It has been identified via resistance to pepsin, sensitivity to purified collagenase, and its immunoreactivity with several antibodies [203]. It shows an unusual corkscrew distribution pattern related to the dentin enamel interface [204]. As a single structural protein, it has intra- and extra-cellular consequences, resulting in inflammatory processes which can promote keratinocyte-driven tissue destabilization and progressive fibrosis [205].

## 10. Collagen VIII

It is a nonfibrillar, a short-chain collagen, and the main components of Descemet’s membrane [206], which is basically synthesized by endothelial cells, and can be found in cartilage, muscles, lung, liver, brain, and heart [207]. It was first discovered as an extracellular protein in bovine aortic endothelial cell cultures which is composed of α1 and α2 collagen chains [206,207]. It can be found in large fibrotic vessels of angiomas and proliferating vessels of brain tumors with signaling and structural properties [207,208]. Its signaling properties originate from vastatin, which is a non-collagenous C-terminal globular NC1 [207,208]; moreover, it is an important component of blood vessels [209]. It has important functions in treatment of systemic sclerosis, angina pectoris, chronic obstructive lung disease, and different cancers [210]. It can negatively regulate elastin, and it can also increase the content in carotid arteries and elastin synthesis [211]. It is significantly upregulated in different cancer types [210,211], with important function in glial scar formation during the repair process by astrocytes [212]. Collagen VII is important for the maintenance of vessel wall elasticity and integrity [213,214].

## 11. Collagen X

Collagen type X is strongly expressed in hypertrophic chondrocytes, and it is a short-chain collagen [161]. It is expressed particularly in the growth plate of long bones [161]. Metaphyseal chondrodysplasia type Schmid (MCDS) is caused by mutations in the human collagen X gene [162]. It is a network-forming collagen which is basically expressed in hypertrophic chondrocytes in the cartilage of the hypertrophic zone of the growth plate [215]. It is active in human lumbar intervertebral discs, and it is produced by hypertrophic cartilage undergoing endochondral ossification [216]. Its biomarkers are shown to be elevated in rheumatological disorders influencing cartilage and bone, and of course, in cancer treatment [215,216]. It appears in the matrix of the hypertrophic zone [215,216,217,218].

## 12. Collagen XI

It is a minor fibrillar collagen which is extensively distributed in the lung, placenta, skeletal muscle, trabecular bone, tendon, trachea, testis, articular cartilage, and the neuroepithelium of the brain [219]. It can regulate fibrillogenesis by keeping the spacing and diameter of type II collagen fibrils [220]. It can also work as a nucleator for the fibrillogenesis of types II and I collagens [219,220], and it has various isoforms such as α1(XI)α2(XI)α3(XI), as well as XI and V hybrids such as [α1(XI)]_2_α2(V) [219,220]. COL11A1, COL11A2, and COL2A1 can encode different three α-chains of collagen type XI, namely α1(XI), α2(XI), and α3(II), respectively [221], and this collagen type plays an important role in skeletal morphogenesis and fibril formation [222]. Mutations in type XI collagen are linked with Marshall syndrome, Stickler syndrome, Weissenbacher−Zweymuller syndrome, otospondylomegaepiphyseal dysplasia deafness, and fibrochondrogenesis [221,222]. It is also significantly linked with cancer-associated fibroblasts and cancer progression [223], and it can also bind dermatan sulfate, heparan sulfate, and heparin [224]. The α1 chain of a minor human collagen, type XI, is not discovered in normal stroma, but it is overexpressed in some tumor stroma, and its detection may need appropriate probes [159]. Lorenzo-Gomez et al. [160] found that the detection of the α1 chain of human collagen XI was possible in cell lysates, which was also admitted by aptacyfluorescence. Its biomarkers have shown high capability as prognostic and diagnostic markers in cancer diseases [225], and it can regulate mechanical properties and acquisition of fibrillar structure [224,225]. As the ECM component, it shows high activity in increasing the inhibiting and reducing the degradation of the cartilage matrix [224,225]. It can regulate the stroma surrounding breast cancer, and it may help predict which women are at risk of having lymph node metastases [226]. It is present in the nucleus pulposus and fibrosus of intervertebral discs [226,227]. Stromal collagen is associated with invasive recurrence in the breast ductal carcinoma in situ (DCIS) and is an important marker to predict the response to radiotherapy [228], and type XI collagen genes can be considered as important markers for chondrogenic tumors [229,230]. It can be considered as a locus for fibrochondrogenesis and reveal the possible phenotypic manifestations among carriers [231]. In addition, it can increase the potential of distinguishing the cancer-associated desmoplastic stroma which is linked with misplaced adenomatous mucosa [232]. It is important for the development and integrity of the skeleton because of mutations in the genes [233].

Unlike type II collagen, the structure of collagen type XI has been of interest because the proteolytic processing of the amino-terminal domain is very slow and sometime after processing is completed, and some portion of the domain is retained [163]. Collagen type XI plays a role in the control of fibril diameter [164], and sometimes, mutations in its genes have been reported to cause multiple epiphyseal dysplasia (MED) [163,164,165,166]. It can also interact with type IX and type II collagens to form the meshwork of collagen fibrils, which gives cartilage its notable tensile strength [167]. It is also reported that type XI and type V collagens are not separate collagen types but are part of a larger collagen family in which chains of both collagens participate in the formation of different native molecules [166,167]. The specific locations of type XI and V collagens in cartilaginous tissue can play different functional roles for these two components in the tissue [166,167].

## 13. Collagen IX

Collagen IX is synthesized as short and long forms which lack or contain, respectively, a 27 kDa non-collagenous (NC) 4 domains at the N-terminus of the α1(IX) chain of the molecules [168,234,235], it is an important structural component of the extracellular matrix of connective tissues [169]. Moreover, it is important for the normal structural integrity of the tissue [170]. It is a fibril-associated collagen with interrupted triple helices [233]. It is present in adult articular cartilage, chondrocytes of growth-plate cartilage, the inner ear, and intervertebral discs [234]. It can contribute to the stabilization of the fibrillar collagen network in the cartilage matrix [234,235]. Its absence may lead to early developmental, biomechanical, and structural changes in the spine, which lay the ground for disc degeneration in aged mice [235], and it is an important part of the articular cartilage [236,237]. Its mutations may induce abnormal integrity of collagen fibers in the tectorial membrane [237], and it plays an important role in the tectorial membrane in the auditory system [238]. The interaction between biglycan and collagen IX can be considered as the weak link in the cartilage collagen architecture [238].

## 14. Collagen XII

Type XIII collagen molecules are believed to be composed of four non-collagenous domains (NC1-4) and three collagenous domains (COL1−3), with two located at the N- and C-terminal ends of the polypeptide and two of them separating the collagenous domains [161,241]. It is a fibril-associated collagen with interrupted triple helices [239], and it consists of alpha chains which are nearly 90 kDa larger than the 200 kDa alpha chain [240]. It can be found in the interstitial matrix and is an ingredient of human skeletal muscle [241]. It is important to stabilize the joint extracellular matrix in humans and mice [242], and it can interact with tenascin, fibromodulin, COMP, and decorin [243]. It has function in regulating corneal stromal transforming growth factor (TGF)-β activation and latency [244]. It can prevent the formation of atherosclerotic lesions, and it can also stabilize the vascular structure [243,244]. Collagens XIV and XII are both associated with muscle metabolism [243,244], and this association occurs regardless of breeds [245]. It is important to attain wound closure and scar maturation [246], and it is a key element which can control growth factor availability, improve the orchestration of a proper skin matrix structure and regulate cellular function and composition [246,247]. Type XIV and XII collagens produced by tenocytes represent minor collagenous components of the tendon [247,248,249].

## 15. Collagen XIII

Collagen XIII is a nonfibrillar, type II transmembrane collagen which belongs to a subgroup of collagens called membrane-associated collagen with interrupted triple helices [250]. It includes a short N-terminal cytosolic domain, a largely collagenous ectodomain, a transmembrane domain, and exists as a soluble form because of its ectodomain shedding [251]. It can bind to proteins like α1β1 integrin, type IV collagen, nidogen-2, vitronectin, perlecan, and fibronectin, and its expression decreases toward adulthood and becomes more pronounced during postnatal and development growth [250,251]. The complete primary structure of the mouse type XIII collagen chain was shown by cDNA cloning [252], and it is significantly expressed in cells producing connective tissue, during corneal wound healing, with higher expression in certain tumors and renal fibrosis [253]. It is important for the normal function and structure of neuromuscular synapses [253,254], and its loss may influence maturation of both the pre- and postsynaptic specialization of the neuromuscular junctions [254,255,256]. Its overexpression can induce the development of massive bone overgrowth because of an increased osteoblast differentiation capacity [257,258]. It is also reported that the most important collagen ectodomain which is in triple helical conformation is type XIII collagen [259].

## 16. Collagen XIV

Collagen XIV belongs to the subclass of fibril-associated collagens with interrupted triple helices [169,170], and it is strongly present in the native human bone marrow, as shown by immunofluorescence staining and immunoblotting with an affinity-purified antibody [171]. Both collagens XIV and XII localize near the surface of banded collagen fibrils [172]. Collagen XIV is a fibril-associated collagen with interrupted triple helices which can be found mainly in articular cartilage, cornea, tendon, and the skin [260], and it can adhere to fibrillar collagen to regulate fibrillogenesis by limiting the collagen fibril diameter [261]. It can prevent the lateral fusion of adjacent fibrils [261,262], and it can bind to the dermatan sulfate chain of decorin and to the heparan sulfate chain of perlecan [263]. Its expression is highest during development, but it is also co-distributed with type I collagen in adult tissues [264,265,266]. It consists of three α1(XIV) chains [264], and each α1(XIV) chain is composed of two collagenous domains, namely COL2 and COL, with three non-collagenous NC1-NC3 domains [264]. It is present in tissue areas with high mechanical stress, showing that it provides important mechanical characteristics to tissues [265]. It is reported that increased type XIV collagen expression has been indicated in the progression of lung fibrosis and metastatic colon cancer [265]. It plays a significant regulatory role in early stages of collagen fibrillogenesis with tissue differences [266], and it has an important function in corneal development with an important role in the adult cornea [265,266].

## 17. Collagen XV

Human collagen XV is a homotrimer with α chains consisting of 1363 amino acid residues, including a highly interrupted collagenous domain of 577 amino acids. It shares structural homology with collagen XVIII, with the highest similarity in the C-terminal non-collagenous domain, known as the endostatin domain [267]. It is a nonfibrillar basement membrane-associated collagen [268]. It is produced mainly by endothelial cells, muscle cells, and fibroblasts, and it is basically located in the basement membrane zones of cardiac, microvessels, and skeletal myocytes [269]. Collagens XVIII and XV are very similar, and both are the members of the multiplexin collagen family [269]. The maximum level of sequence homology between types XVIII and XV is located in the NC1 domain [269]. It is reported that collagen XV may have important function in the invasiveness of tumors due to its ductal carcinomas and adenocarcinomas, and it may inhibit the migration and adhesion of fibrosarcoma cells when available in fibronectin-containing matrices [270]. Its expression is widespread in the basement membrane zones of various internal organs, such as testis, ovary, skeletal, heart, kidney, adrenal gland, and placenta [271], and the human gene for the α1 chain of type XV collagen is around 145 kilobases in size with around 42 exons. The NC1 domains of both types are organized into an N-terminal trimerization domain [272]. The high stability, solubility, expression level, and its small size of its trimerization domain make it appropriate for engineering homotrimeric antibodies for cancer therapy and detection [273,274]. It has notable functions in the neuromuscular and cardiovascular systems [274]. It can enhance adhesion of cells to collagen L, and its gene and its human counterpart can be found in the chromosomal segments with conserved syntenies [274,275].

## 18. Collagen XVI

Nonfibrillar collagens such as XXV, XVII, and XIII appear to localize at the cell surface, and they have transmembrane domains [276]. It is part of the family of fibril-associated collagens with interrupted triple helices [277]. It is synthesized by different cell types such as myofibroblasts, chondrocytes, smooth muscle cells, keratinocytes, dendrocytes, and fibroblasts [277]. It is observed that the collagen type XVI is regulated by TGF-β and bFGF in a manner very similar to the regulation of collagen I in arterial smooth muscle cells and human dermal fibroblasts [278]. Collagen XVI can also act as a substrate for invasion and adhesion of connective tissue tumor cells, altering the cell−matrix interaction and induce a proliferative tumor phenotype by increasing an early S-phase entry [279]. It is expressed in different tissues such as kidney, arterial walls, lung, intestine, heart, cartilage, and skin [280]. Its major functions are anchoring microfibrils to the basement membrane, organize the extracellular matrix by focal adhesions and collagen fibrils and mediate intracellular signaling which can influence cell invasiveness, proliferation, adhesion, and the formation of focal adhesions [281]. It is integrated into particular fibrillin-rich microfibrils in skin which lacks an amorphous elastin core [282], and it is a component of small heterotypic D-banded fibrils, basically found in the territorial matrix of chondrocytes in cartilage [282]. Collagen XVI may show collagenous constituents in the extracellular matrix and may play an important role in the structural integrity of different tissues [283], and it is a transmembrane protein which mediates skin homeostasis [283].

## 19. Collagen XVII

It is a hemidesmosomal anchorage molecule of basal keratinocytes which increases stable epidermal−dermal adhesion [284], and it has function in epithelial hemidesmosomes of colonic mucosa, skin, brain, cornea, placenta, and kidney with different binding partners [285]. COL15 is the largest collagenous domain of type XVII, which has been reported as a cell adhesion domain [284,285], and it keeps the linkage between extracellular and intracellular structures [286]. Its ectodomain, part of its transmembrane molecule, includes a series of collagen-like repeats which is related to homotrimeric structures [286], and the intracellular domain binds to bullous pemphigoid antigen, integrin α6β4, and plectin [287]. As a structural transmembrane component of the hemidesmosomes, collagen XVII keeps stable dermis−epidermis adhesion and is traditionally linked with the pathogenesis of blistering skin disease [288]. It is abundant in the epidermis, which is an ectodermal derivative [289], and it consists of three 180 kDa α1 (XVII) chains, each containing an intracellular N-terminal domain with a short transmembrane stretch [289]. One of the main characteristics of collagen XVII is the constitutive release of its collagenous ectodomain from the cell surface [290]. Collagen XVII directly binds to collagen IV at the basement membrane zone in both oral mucosa and skin [291]. As it is a transmembrane hemidesmosomal protein of basal keratinocytes, the changed expression of collagen XVII is linked with melanoma progression [292]. Mutations in type XVII collagen can induce epithelial recurrent erosion dystrophy disease with recurrent corneal erosions and junctional epidermolysis bullosa with skin blistering [293], and collagen XVII plays a role in various cancers in relations to proliferation, migration, and overall survival of the patients [293,294]. It is an autoantigen in bullous pemphigoid, a blistering skin disease [293,294], and it is discovered to be linked with the maintenance of EMT phenotypes and metastasis capability in lung cancer stem cells [295]. Its integrin-β1 activity can increase contact following collective invasion and is localized to the intercellular site in a cancer cell population [296]. In addition, COL17A1 is highly expressed in hair follicle stem cells and involved in hair-follicle-associated pluripotent (HAP) stem cell differentiation [297].

## 20. Collagen XVIII

Collagen XVIII is in the regression of vasa hyaloidea propria, while collagen XV plays an important role in the tunica vasculosa lentis regression procedure [298]. On the basis of reports on fragments, collagens XVIII and XV have a similar binding repertoire for extracellular matrix proteins, but they have differences in basement membrane zones and the immunohistological localization in vessel walls [298]. It is known by three variants N termini, a C-terminal antiangiogenic domain known as endostatin, and an interrupted collagenous domain [299]. It is reported that mutations in human collagen XVIII may induce occipital encephalocele in Knobloch syndrome, macular abnormalities, and vitreoretinal degeneration [300]. Collagen type XVIII was found to be a ubiquitous basement membrane ingredient, occurring prominently at epithelial and vascular basement membranes throughout the body [300,301]. It possesses features of proteoglycans and collagens which are localized in different basement membrane zones, and it can inhibit tumor growth and angiogenesis [302]. Its absence may cause distinct ultrastructural defects of nephrons [302,303]. Collagen XVIII isoforms have various spatial and temporal expression patterns during renal development [303,304], and its isoforms vary in their N-terminal domains; however, they have a common C-terminal anti-angiogenic endostatin domain [305]. The long isoform is available in the liver, and the short isoform is available in epithelial and vascular basement membrane structures [305]. It plays an important role in maintaining basement membrane integrity and has positive function in eye development [306]. It is abundant in basal laminae of epidermis, retina, cardiac, pia, striated muscle, lung, blood vessels, and kidney [306], and it can influence the skin structural characteristics, resulting in decreased wrinkles, increased surface homogeneity, and improved skin elasticity [307].

## 21. Collagen XIX

Collagen type XIX is a collagen which is linked with the basement membrane zone [300,301], and it can bind to αvβ3 integrin, reducing the phosphorylation of proteins which are active in the FAK/PI3K/Akt/mTOR pathway [301]. It is a member of the fibril-associated collagens with the interrupted triple helices (FACIT) family [308]. It can organize the ECM through its ability to manage as a cross-bridge between other ECM molecules and fibrils [309]. It is a homotrimer composed of three α1 chains and is expressed in mesenchymal, neuronal, vascular, and epithelial basement membrane zones in spleen, skin, skeletal muscle, prostate, placenta, liver, kidney, colon, and breast [310]. It is also active in the development of muscle tissue of the heart and esophagus as well as the brain [310].

## 22. Collagen XX

It is assigned to the fibril-associated collagens with the interrupted triple helices collagen subfamily because of its similarities with type XIV and XII collagens [311]. It consists of a von Willebrand factor A domain, six fibronectin type III repeat domains, two triple-helical domains, and a laminin G-like domain [312].

## 23. Collagen XXI

It belongs to the family of fibril-associated collagens with interrupted triple helices [313], and it serves as a molecular bridge between different extracellular matrix proteins and collagen fibrils [314]. It is expressed in lymph nodes, pancreas, lung, kidney, skeletal muscle, jejunum, stomach, placenta, and heart [314].

## 24. Collagen XXII

As the minor collagen, it belongs to the family of fibril-associated collagens with interrupted triple helices [315], and it is usually expressed at the basement membrane zones of tissue junctions in cartilage, heart, skin, and muscle [316]. It helps stabilize the junctions by binding to integrins α11β1 and α2β1 [317]. It plays an important role in cancer treatment, keeping vascular stability, and supporting tissue development [316,317].

## 25. Collagen XXIII

It is a type II transmembrane collagen [318], which can be identified in mouse tissues and healthy human tissues of tendon, skin, cornea, and lung [318]. It plays an important role in the polarization of epithelial cells and keeps the formation of cell−cell contacts [319]. It plays an important role in diagnosis of certain kinds of cancer [320].

## 26. Collagen XXV

Collagen XXV belongs to the membrane collagen subgroup, and it is basically expressed in the neurons of the brain, different tissues of the eye testis, and the heart [321]. Collagen XXV is vital during early muscle and brain development.

## 27. Collagen XXVI

Collagen XXVI includes a collagenous domain in its structure; however, it does not fit squarely within any of the collagen family subgroups [322]. It has a connection with modeling and generation of tissue, and its primers are mainly formed from the intermolecular disulfide bonds between NC1 regions [322]. No mutations or biomarkers have been found so far for this protein [322].

## 28. Collagen XXIV

Collagens XXVI, XXIV, XXVII, XXVIII, and XXIX are fibril-associated collagens with interrupted triple helices (FACIT), which associate with the surface of the collagen fibrils, and modify their interactive characteristics [323]. Collagens support soft tissues and cardiovascular components [324]. Collagen XXIV is a fibrillar collagen including three non-collagenous domains and two collagenous domains [325]. It is commonly expressed in the formation of bone, as well as the ovary, testis, lung, liver, spleen, kidneys, muscle, and brain [326]. It can be as a marker of osteoblast differentiation [327], and it consists of a long triple helical domain flanked by typical propeptide-like sequences [328]. It is also suggested that collagen XXIV, as an ancient molecule, may play an important role in the regulation of type I collagen fibrillogenesis [329].

## 29. Collagen XXVII

It is reported that collagens XXVII and XXIV are likely to form distinct homotrimers [324,330]. Collagen XXVIII is commonly found in low levels in healthy lung tissue; however, its expression is enhanced in a bleomycin-induced lung injury model, and it plays an important role in damage repair [331]. It is a fibrillar collagen, which has a different molecular structure than other fibrillar collagens [332,333]. In its structure, it has no N-terminal telopeptide-like region and has a minor triple-helical domain [334]. Steel syndrome is a disease that is definitively connected to mutations in the type XXVII collagen gene [335,336]. Collagen type XXVII has significant function during the calcification of cartilage and during the transition from cartilage to bone [336].

## 30. Collagen XXVIII

It is reported that collagens XXVII and XXIV are likely to form distinct homotrimers [324,330]. Collagen XXVIII is commonly found in low levels in healthy lung tissue; however, its expression is enhanced in a bleomycin-induced lung injury model, and it plays an important role in damage repair [331]. Its structure is similar to collagen type VI [337]. It is located in dorsal root ganglia and in peripheral nerves surrounding most non-myelinating glial cells [337]. It can be found in skin calvaria, and it is also located in murine lungs related to basement membranes [338].

## 31. Collagen XXIX

Collagen XXIX belongs to the group of collagens consisting of von Willebrand factor type A domains [339], and it plays a significant role in protein−ligand interactions for the organization of cell adhesion and tissue architecture [340].

## 32. The Main System of Recombinant Collagen Expression and Its Expression Research Status

The application of recombinant human collagens in medicine and research, as well as cosmetic, food, and drug industries, offers a promising alternative to the application of collagen materials. Due to its considerable structure, collagen has exceptional degradability, hemostasis, and biocompatibility characteristics, which is widely applied tissue repair in cartilage, bone, teeth, skin, cornea, neurons, and different other aspects [341]. It stands as the predominant protein in vertebrates and includes around 25% of the total proteins of vertebrates [342], which is also characterized by its fibrous structure, and its capability to break down in an environmentally friendly manner [341,342,343,344,345,346]. The advantage of collagen synthesis has been shown by different studies, and researchers have reported less joint pain perception after collagen peptide application in patients with osteoarthritis [347,348,349]. Collagen peptides have been found to show significant physiological functions with a positive effect on health, and various studies have shown the recovery of lost cartilage tissue, an improvement in skin elasticity, strengthened tendons and ligaments, reduced activity-related joint pain, increased bone mineral density in postmenopausal women, and enhanced lean body mass in premenopausal women and elderly men [350,351,352]. Important types of recombinant human-like collagen and natural collagen are allergenicity, affinity, production technology, biological activity, security, and purity. The expression systems of recombinant collagen usually fall into the following groups: microbial expression systems including *E. coli* (prokaryotic), eukaryotic expression systems, animals, and plant expression systems [347,348,349,350]. *E. coli* expression currently stands as the foremost prokaryotic cell expression system utilized in the production of recombinant proteins, and its advantages include a well-defined genetic background, an exhaustive range of commercial vectors and strains, an intricate regulatory mechanism, cost-effectiveness, a short growth cycle, and exceptional expression effectiveness. However, there are also some deficiencies related to the *E. coli* expression system, such as the inability to glycosylate proteins, low biological activity, formation of insoluble inclusion bodies because of insufficient secretion capacity, and the arduous task of eradicating endotoxins it generates [350,351,352,353,354,355,356,357,358,359,360,361,362,363,364]. The yeast expression system shows different benefits including the absence of endotoxin, the ability for large-scale high-density culture, simplicity in operation, and cost-effectiveness [351,352,353]. Animal and plant expression systems commonly use host cells including insect cells, mammalian mouse cells, and silkworm cells for animal expression systems, and the advantage of the insect expression system is that the recombinant collagen retains its complete protein structure and biological activity, can accept the insertion of large fragments of foreign genes and enables extracellular protein expression. However, the disadvantages are also considerable such as glycosylation which is different from that of mammals, high cost, and limited protein expression [353,354,355].

Some examples of experimental protein replacement techniques with the application of recombinant collagens are collagen VII for dystrophic epidermolysis bullosa in which intravenous or intradermal delivery of recombinant collagen VII into mice was checked [353,354], and systemic delivery for collagen IV for Alport syndrome [354]. Although significant progress has been made to produce and design recombinant collagens with collagen-derived constructs and native structures, the proteins have not advanced very well in the clinical marketplace, as there is no clear consensus on a system for large-scale recombinant collagen production that meets the regulatory factors responsible for approval in commercial clinical applications. Additionally, there is no consensus on the most related forms of recombinant collagens needed in the market, and there is still no recognizable leading product which could attract the attention of the market. While tissue engineering is still a promising application for recombinant collagen variants, its potential has not been fully realized in novel recombinant collagen-based materials in different ways [353,354]. In tissue engineering applications, recombinant collagen biomaterials have been widely studied for skin regeneration because of their natural abundance of collagen in the dermis, as collagen hydrogels promote wound healing by stimulating cell proliferation, migration, angiogenesis, and collagen deposition [351,352,353,354].

Various tissue engineering techniques for recombinant human collagens focus on stroma regeneration, which can promote skin regeneration, support cell differentiation, and proliferation. It can be used to construct different tissue scaffolds for wound treatment, which can provide unique biocompatibility and cell adhesion and increase wound healing; for 3D printing which has potential to create personalized implants and can create complex and patient-specific structures. In orthopedics, it is appropriate for immune response, stimulating bone growth, is applied for bone tissue techniques and offers high bioresorbability and biocompatibility [352]. Important points of physicochemical identifications of recombinant collagens are shown in Table 3. Important points of recombinant human-like collagens and natural collagens are shown in Table 4. Various tissue engineering techniques for recombinant human collagens are shown in Table 5.

Bio-production of recombinant collagens still suffer from high cost and low yield, and the products are unable to obtain the same post-translational changes seen in native collagens, which makes current recombinant collagens both non-biological and expensive. Therefore, new and trustable bio-manufacturing techniques are needed to make economical large-scale manufacturing of recombinant collagens suitable for future studies [353]. It is also important to study and design a versatile platform to analyze tissue-specific cellular interactions by applying collagen type I scaffolds with highly tunable biophysical characteristics, as the kinetics of collagen fibrillogenesis are stimulated via a combination of varied pH and shear rate during neutralization to obtain a broad range of fibril diameter, porosity, anisotropy, and storage modulus. The function of each of these properties in guiding vascular, bone, and muscle cell types is well recognized and evaluated in vitro during the generation of various distinct musculoskeletal engineered constructs.

## 33. Necessary Factors for the Formation of Collagen Structure: Proline Hydroxylation and Glycosylation

The major common factor of all collagens is their noticeable structural element which is the collagen triple helix [354], also known as a triple-helical domain [354]. The tiple-helical domain of type I collagen has a semi-rigid rod-like structure of about 1.5 nm width and 300 nm length [354]. It is important to consider the dual functions of collagens in the ECM, acting as both signaling molecules in matricellular communications and structural components [354,355]. Its defining characteristic is an elegant structural motif in which three parallel polypeptide strands, in a left-handed, polyproline II-type helical conformation, coil about each other with a one-residue stagger to form a right-handed triple helix.

The post-translational modifications of a collagen single chain start with hydroxylation or proline and lysine residues, which thermally stabilizes the triple helix [355], and after that, some of the formed hydroxylysine (Hyl) residues are glycosylated. Then, the unique glycosylation of collagen includes linking β-D-galactopyranose via β-linkage to Hyl and adding glucopyranose to the C2 position of galactopyranose by α-linkage [356]. It is also reported that collagen glycosylation was associated with autoimmune diseases like systemic sclerosis, rheumatoid arthritis, and cancer [356].

Higher stability of the collagen structure can be related to the capability of new dimethyl sulfoxide (DMSO) to simultaneously change both resin components and biological components within the hybrid layer [357]. Proline hydroxylation is extensively found post-translational modification with collagen being the pre-eminent sample [356,357], and the hydroxylation of proline is commonly discovered in conotoxins which are the main component of many samples [358,359,360]. The position of glycosylated hydroxylysine, which is clarified by comprehensive liquid chromatography (LC)/MS analysis, can provide clear insights into the physiological function of the various modifications. The lysine (Lys) hydroxylation pattern of collagen type I produced by fibroblasts is domain-specific within the collagen molecule, which is an appropriate method to characterize the cell phenotypes in pathological/normal connective tissues [357,358,359,360]. The new sustainable technique for solubilizing collagen provides a direct method for preparing active and essential low-molecular-weight collagen peptides directly from collagen in a single step, which can enable the sustainable dissolution of collagen, exposing aromatic amino acid residues with obvious antibacterial activity [360,361]. Collagen gradient membranes showed a dense outer layer and a loose inner layer, as membranes had good porosity, strong mechanical properties, and hydrophilicity [360,361]. In another experiment, N-hydroxysuccinimide-activated suberic acid (NHS-SA)-crosslinked assembled collagen fibers (NACFs) implant showed promising results in collagen regeneration, which present its importance in advancing skin rejuvenation therapies. The structure of collagen may regulate affinity and the extent of the binding under flow of plasma constituents such as the Willebrand factor. Mutations can also influence collagen fiber structure, formation, as well as function, which can result in different bone pathologies, highlighting the significance of collagen in maintaining healthy bone tissue.

It is believed that fibril formation happens more readily on more hydrophobic surfaces, which is attributed to a higher mobility of individual molecules adsorbed on more hydrophobic substances [359,360,361]. The rate of fibrillogenesis of the collagen−DNA complex associates to the DNA structure, and the spatial distribution of collagen and DNA in the complexes shows that the characteristic collagen−DNA interaction relates to the DNA forms [361,362]. It is also confirmed that collagen undergoes conformational alterations related to pH changes during disease as it is the main component of the extracellular matrix, and more research on this topic can help to better understand how pH changes can be modulated to restore healthy collagen characteristics [360,361,362]. Post-translational modifications of collagen, namely *N*-glycosylation, the *O*-glycosylation of Hyl, lysine and proline hydroxylation, the oxidative deamination of Hyl and Lys residues in the telopeptide domains, and following inter- and intramolecular covalent cross-linking are important characteristics of collagen are important characteristics of collagen [360,361,362]. Moreover, the hydroxylation of proline in positions 3 and 4 is essential for the stability of the triple-helical structure, while the hydroxylation of lysine residues is needed for subsequent glycosylation [361,362,363,364].

## 34. Conclusions

Collagens are divided into two groups: fibrillar collagens, such as collagens I, II, III, V, XI, XIV, and XXVII, and non-fibrillar collagens, such as collagens IV, VII, VI, XXVIII, XXIX, VIII, X, XIII, XVII, XXIII, XXV, IX, XII, XIV, XVI, XIX, XX, XXI, XXII, XXVI, XV, and XVIII. The most abundant structural protein in vertebrates is collagen type I, which is a heterotrimeric molecule composed of one α1 chains and one α2 chain, forming a long uninterrupted triple helical structure with short non-triple helical telopeptides at both the C- and N-termini. The most ubiquitous and abundant collagen macroaggregates are the highly ordered banded fibrils made of fibrillar collagen, namely collagens XI, V, I, II, and III, which share a common structure, i.e., a long triple helical domain. The significance of collagen types XVIII, XV, and XIII, known solely on the grounds of their repetitive Gly-X-Y sequence, can be only evaluated from a genetic analysis of mutations in the respective genes. The location of collagen I is in vascular ligature, cornea, tendon, bone, and skin; collagen II is in vitreous body, cartilage, and gristle; collagen III is available in reticulate, intestine, uterus, skin, and vessels; collagen V is in hair, cornea, placenta, skin, bones, and cell surfaces; collagen IV is in capillaries and forms basal lamina; collagen VI is in cornea, gristle, vessels, bones, and skin; collagen VII is in amniotic fluid, skin, bladder, and mucous membranes; collagen VIII is in gristle, vessels, bones, brain, kidneys, skin, and heart; collagen X is in gristle; collagen XI is in intervertebral disc and gristle; collagen IX is in gristle, vitreous body, and cornea; collagen XII is in tendons, skin, and gristle; collagen XIII is in skin, endothelial cells, heart, eye, and skeletal muscles; collagen XIV is in gristle, skin, tendons, bones, eye, nerves, and vessels; collagen XV is in placenta, kidneys, testicles, skin, ovaries, heart, and capillary vessels; collagen XVI is in kidneys, smooth muscle, heart, and skin; collagen XVII is in skin; collagen XVIII is in lungs, liver, and kidney; collagen XIX is in prostate gland, placenta, spleen, kidneys, liver, and skin; collagen XX is in corneal epithelium; collagen XXI is in vessels, skeletal muscles, placenta, kidneys, heart, and stomach; collagen XXII is in tissue connections; collagen XXIII is in metastatic carcinogenic cells; collagen XXV is in testicles, brain, heart, and eye; collagen XXVI is in ovaries and testicles; collagen XXIV is in cornea and bones; collagen XXVII is in gristle; collagen XXVIII is in nervous system cells; and collagen XXIX is in skin. The most widespread and abundant family of collagens is presented by fibril-forming collagens, while types V and I collagen fibrils play important roles in the structural backbone of bone, types XI and II collagens usually contribute to the fibrillar matrix of articular cartilage, and type IV collagens with their significant flexible triple helix assemble into mesh-works blocked to basement membranes. Fibril-associated collagens such as collagens XIV, XII, and IX apparently have a function in regulating the diameter of collagen fibrils, and type X and VIII collagens from hexagonal networks. It is important to consider the point that all features of collagens are a triple helical conformation composed of repeats of the G-X-Y motif, in which proline and lysine usually occur at the y and x positions. During translation in the endoplasmic reticulum, selected lysine and proline residues are hydroxylated by dedicated hydroxylases, therefore yielding hydroxylysine and hydroxyproline. The formation of hydroxyproline is important to stabilize the collagen triple helix and confer its thermal stability at body temperature. Lysyl hydroxylation is active in the formation of covalent inter- and intra-molecular crosslinks, contributing to fibril formation and condensation. Hydroxylysine also acts as an acceptor for the attachment of collagen specific glycans. Microbes’ expression system has a clear genetic background, strong operability, and low cost and is suitable for large-scale production; animal expression system is suitable for the expression of complete macromolecular proteins, which can be secreted and expressed, exhibit high protein activity and undergo correct advanced structure and protein modification such as glycosylation and hydroxylation; the plant expression system has a wide range of host sources and low cost with high security. It is obvious that with technological and scientific progresses, recombinant collagens will play a significant role in the large-scale collagen production, and on the basis of findings, recombinant collagens have special potential for various applications. However, more research is needed for recombinant collagens or combining the recombinant collagen with new biological materials and information to make them effective in a broad spectrum of biological fields.

## Figures and Tables

**Table 1:** Important information on different collagens of animal by-products and extraction methods of collagens.

Animal by-Products Collagen	Collagen Types	Location of Collagen	Collagen Extraction Methods
Fish bones and skin	Type I	Skin, bone, scales, and body	Acid extraction procedure; pepsin-aided acetic acid extraction procedure
	Types I and V	Mantle	Acid extraction procedure; pepsin-aided acetic acid extraction procedure
	Type IV	Tissues	Acid extraction procedure; pepsin-aided acetic acid extraction procedure
	Types I, II, and III	Bell and oral arms	Acid extraction procedure; Pepsin-aided acetic acid extraction procedure
Bovine tendons, body, organs, and skin	Type I and type V	Body	Treatment with a defatting agent at soft temperature; treatment with soft acid and pepsin at low temperature; treatment with acid/alkali or high temperature; treatment with enzymes to break down polypeptide chains collagen; treatment with enzymes followed by isolation of certain bioactive peptides
	Type II	Elastic cartilage	treatment with a defatting agent at soft temperature; treatment with soft acid and pepsin at low temperature; treatment with acid/alkali or high temperature; treatment with enzymes to break down polypeptide chains collagen; treatment with enzymes followed by isolation of certain bioactive peptides
	Type III	Muscles, organs, and arteries	Treatment with a defatting agent at soft temperature; treatment with soft acid and pepsin at low temperature; treatment with acid/alkali or high temperature
	Type IV	The Layer of skin	Treatment with acid/alkali or high temperature; treatment with enzymes to break down polypeptide chains collagen; treatment with enzymes followed by isolation of certain bioactive peptides
Porcine skins	Types I, II, and III	Skin	Treatment with a defatting agent at soft temperature; treatment with soft acid and pepsin at low temperature; treatment with acid/alkali or high temperature
Poultry cartilages, skin, and bone	Types I and III	Skin	Enzyme hydrolysis extraction for skin; acid-soluble extraction for bone; alkali-soluble extraction for bone
	Type II	Cartilage	Enzyme hydrolysis extraction for skin; acid-soluble extraction for bone; alkali-soluble extraction for bone
	Types I and II	Bone	Enzyme hydrolysis extraction for skin; acid-soluble extraction for bone; alkali-soluble extraction for bone

**Table 2:** The collagen proteins family and associated disorders.

Collagens	Type	Location
Fibrillar collagens	Collagens I	Bone, skin, cornea, tendon, vascular ligature, and organs
	Collagens II	Vitreous body, cartilage, and gristle
	Collagens III	Reticulate, commonly found together with collagen type I skin, vessels, intestine, and uterus
	Collagens V	Skin, cell surfaces, cornea, placenta, skin, and hair
	Collagens XI	Intervertebral disc, and gristle
	Collagens XIV	Gristle, skin, tendons, bones, eye, nerves, and vessels
	Collagens XXVII	Gristle
Nonfibrillar collagens	Collagens IV	Forms basal lamina, the epithelium-secreted layer of the basement membrane, and capillaries
	Collagens VII	Umbilical cord, amniotic fluid, skin, bladder, and mucous membranes
	Collagens VI, XXVIII, and XXIX	Collagen VI can be found in cornea, gristle, vessels, bones, and skin Collagen XXVIII can be found in nervous system cells. Collagen XXIX can be found in skin.
	Collagens VIII, X	Collagen VIII can be found in gristle, vessels, bones, brain, kidneys, skin, and heart. Collagen V can be found in gristle.
	Collagens XIII, XVII, XXIII, and XXV	Collagen XIII can be found in skin, endothelial cells, heart, eye, and skeletal muscles. Collagen XVII can be found in skin. Collagen XXIII can be found in metastatic carcinogenic cells. Collagen XXV can be found in testicles, brain, heart, and eye.
	Collagens IX, XII, XIV, XVI, XIX, XX, XXI, XXII, XXVI, and XXIV	Collagen IX can be found in the gristle, vitreous body, and cornea. Collagen XII can be found in the tendons, skin, and gristle. Collagen XVI can be found in the kidneys, smooth muscle, heart, and skin. Collagen XIV can be found in gristle, skin, tendons, bones, eye, nerves, and vessels. Collagen XIX can be found in the prostate gland, placenta, spleen, kidneys, liver, and skin. Collagen XX can be found in corneal epithelium. Collagen XXI can be found in vessels, skeletal muscles, placenta, kidneys, heart, and stomach. Collagen XXII can be found in tissue connections. Collagen XXVI can be found in ovaries and testicles. Collagen XXIV can be found in cornea and bones.
	Collagens XV and XVIII	Collagen XV can be found in placenta, kidneys, testicles, skin, ovaries, heart, and capillary vessels. Collagen XVIII can be found in lungs, liver, and kidneys.

**Table 3:** Survey on physicochemical identifications of recombinant collagens.

Physiochemical Items	Identified Methods	Requirements
Visible foreign substance	Light inspection	No obvious foreign substance
Appearance	Visual identification	White/like-white powder or sponges in the solid state; white/yellow or transparent liquids or gels
Moisture content	Thermogravimetry analysis	Meeting the technical requirement
Solubility	Visual identification	The solubility should be elaborated and characterized.
pH	pH meter	Meeting the technical requirements
Residue on ignition	High-temperature burning	Meeting the technical requirements
Quantity	-	Meeting the technical requirements
Thermal stability	Differential scanning calorimetry	No gelation of visible floc
Dynamic viscosity	Rotational viscometer method	Meeting the technical requirements

**Table 4:** Important points of recombinant human-like collagens and natural collagens.

Types	Recombinant Human-like Collagen	Natural Collagen
Allergenicity	Low allergy risk	High allergy risk
Affinity	High	Weak
Production technology	Bio-fabrication	Chemical extraction
Biological activity	High	Low
Security	Virus-free	Vulnerable to animal viruses
Purity	Single collagen, fixed composition, and ≤ 95% purity	Mixed collagen and complex composition

**Table 5:** Various tissue engineering techniques for recombinant human collagens [352].

Application	Challenges	Advantages
Stroma regeneration	Control over mechanical characteristics can be challenging.	It promotes skin regeneration
	Possible immunogenicity	It can be used to construct different tissue scaffolds.
		It can support cell differentiation and proliferation.
Wound treatment	Cost of purification and production can be high.	It can be fabricated into different forms.
	Potential for immune response	It can provide unique biocompatibility and cell adhesion.
		It can enhance wound healing.
3D printing	It needs a specialized 3D printing process.	It has potential to create personalized implants.
	It can control over mechanical characteristics.	It can be combined with various materials to enhance its properties.
		It can create complex and patient-specific structures.
Orthopedics	It is appropriate for immune response.	It can stimulate bone growth.
	Mechanical strength can be less than some synthetic materials.	It can offer high bioresorbability and biocompatibility.
		It can be applied for bone tissue techniques.

## Data Availability

Data are contained within the article.

## References

[1] Galdyszynska M., Zwolinski R., Piera L., Szymanski J., Jaszewski R., Drobnik J. (2023). Stiff substrates inhibit collagen accumulation via integrin α2β1, FAK, Src kinases in human atrial fibroblast and myofibroblast cultures derived from patients with aortal stenosis. Biomed. Pharmacother..

[2] Munisso M.C., Saito S., Tsuge I., Morimoto N. (2023). Three-dimensional analysis of load-dependent changes in the orientation of dermal collagen fibers in human skin: A pilot study. J. Mechanic. Behav. Biomed. Mater..

[3] Gronlien K.G., Pedersen M.E., Ronning S.B., Solberg N.T., Tonnesen H.H. (2022). Tuning of 2D cultured human fibroblast behavior using lumichrome photocrosslinked collagen hydrogels. Mat. Today Commun..

[4] Shahrajabian M.H., Sun W., Cheng Q. (2020). Product of natural evolution (SARS, MERS, and SARS-CoV-2); deadly diseases, from SARS to SARS-CoV-2. Hum. Vaccin. Immunother..

[5] Shahrajabian M.H., Sun W., Cheng Q. (2020). Traditional herbal medicine for the prevention and treatment of cold and flu in the autumn of 2020, overlapped with COVID-19. Nat. Prod. Commun..

[6] Shahrajabian M.H., Sun W., Soleymani A., Cheng Q. (2020). Traditional herbal medicines to overcome stress, anxiety and improve mental health in outbreaks of human coronaviruses. Phytother. Res..

[7] Silverman A.A., Olszewski J.D., Siadat S.M., Rubeti J.W. (2024). Tension in the ranks: Cooperative cell contractions drive force-dependent collagen assembly in human fibroblast culture. Matter.

[8] Shahrajabian M.H., Sun W. (2023). The importance of traditional Chinese medicine in the intervention and treatment of HIV while considering its safety and efficacy. Curr. HIV Res..

[9] Shahrajabian M.H., Sun W. (2023). Iranian traditional medicine (ITM) and natural remedies for treatment of the common cold and flu. Rev. Recent Clin. Trials..

[10] Shahrajabian M.H., Sun W. (2023). Five important seeds in traditional medicine, and pharmacological benefits. Seeds.

[11] Patrawalla N.Y., Kajave N.S., Zlbanna M.Z., Kishore V. (2023). Collagen and beyond: A comprehensive comparison of human ECM properties derived from various tissue sources for regenerative medicine applications. J. Funct. Biomater..

[12] Shahrajabian M.H., Sun W. (2024). Multidimensional uses of bitter melon (*Momordica charantia* L.) considering the important functions of its chemical components. Curr. Org. Synth..

[13] Yao L., Blasi J., Shippy T., Brice R. (2023). Transcriptomic analysis reveals the immune response of human microglia to a soy protein and collagen hybrid bioscaffold. Heliyon.

[14] Mukae S., Ogura Y., Hara Y. (2024). Characterization of the collagen network of human cheek skin using ultrasonic microscopy. Ultrasonics.

[15] Carvalho D.N., Gelinsky M., Williams D.S., Mearns-Spragg A., Reis R.L., Silva T.H. (2023). Marine collagen-chitosan-fucoidan/chondroitin sulfate cryo-biomaterials loaded with primary human cells envisaging cartilage tissue engineering. Int. J. Biol. Macromol..

[16] Shahrajabian M.H., Sun W. (2023). Survey on multi-omics, and multi-omics data analysis, integration and application. Curr. Pharm. Anal..

[17] Akoa D.M., Helary C., Foda A., Chaussain C., Poliard A., Coradin T. (2024). Silicon impacts collagen remodelling and mineralization by human dental pulp stem cells in 3D pulp-like matrices. Dent. Mater..

[18] Wang Z., Yang Y., Gao Y., Xu Z., Yang S., Jin M. (2022). Establishing a novel 3D printing bioinks system with recombinant human collagen. Int. J. Biol. Macromol..

[19] Poomrattanangoon S., Pissuwan D. (2024). Gold nanoparticles coated with collagen-I and their wound healing activity in human skin fibroblast cells. Heliyon.

[20] Pu S.-Y., Huang Y.-L., Pu C.-M., Kang Y.-N., Hoang K.D., Chen K.-H., Chen C. (2023). Effects of oral collagen for skin anti-aging: A systematic review and meta-analysis. Nutrients.

[21] Ryabov N.A., Volova L.T., Alekseev D.G., Kovaleva S.A., Medvedeva T.N., Vlasov M.Y. (2024). Mass spectrometry of collagen-containing allogeneic human bone tissue material. Polymers.

[22] Liu X., Li H., Wang T., Yang T., Yang X., Guo K., Hu L., Ming J. (2023). Recombinant humanized collagen type III with high antitumor activity inhibits breast cancer cells autophagy, proliferation, and migration through DDR1. Int. J. Biol. Macrmol..

[23] Shahrajabian M.H., Petropoulos S.A., Sun W. (2023). Survey of the influences of microbial biostimulants on horticultural crops: Case studies and successful paradigms. Horticulturae.

[24] Shahrajabian M.H., Sun W. (2024). Carob (*Ceratonia siliqua* L.), pharmacological and phytochemical activities of neglected legume of the Mediterranean basin, as functional food. Rev. Recent Clin. Trials..

[25] Shahrajabian M.H., Sun W. (2024). Mechanism of action of collagen and epidermal growth factor: A review on theory and research method. Mini-Rev. Med. Chem..

[26] Schwarcz H.P., Nahal H. (2021). Theoretical and observed C/N ratios in human bone collagen. J. Archaeol. Sci..

[27] Shahrajabian M.H., Sun W. (2024). Characterization of intrinsically disordered proteins in healthy and diseased states by nuclear magnetic resonance. Rev. Recent Clin. Trials..

[28] Rosu S.A., Aguilar J., Urbano B.F., Tarraga W.A., Ramella N.A., Longo G.S., Finarelli G.S., Donoso S.A.S., Tricerri M.A. (2023). Interactions of variants of human apoliproprotein A-I with biopolymeric model matrices. Effect of collagen and heparin. Arch. Biochem. Biophys..

[29] Luo X., Liu Y., Zheng C., Huo Q., Liu X. (2021). Development of novel hyaluronic acid/human-like collagen bio-composite membranes: A facile surface modification-assembly approach. Int. J. Biol. Macromol..

[30] Voziyan P., Uppuganti S., Leser M., Rose K.L., Nyman J.S. (2023). Mapping glycation and glycoxidation sites in collagen I of human cortical bone. BBA Adv..

[31] Synytsya A., Janstova D., Smidova M., Synytsya A. (2023). Evaluation of IR and Raman spectroscopic markers of human collagens: Insides for indicating colorectal carcinogenesis. Spectrochim. Acta Part A. Mol. Biomol. Spectroscop..

[32] Pappelbaum K.I., Virgilio N., Epping L., Steen B.V.D., Jimenez F., Funk W., Prawitt J., Bertolini M. (2024). Revealing novel insights on how oral supplementation with collagen peptides may prevent hair loss: Lessons from the human hair follicle organ culture. J. Funct. Foods..

[33] Prade I., Schropfer M., Seidel C., Krumbiegel C., Hille T., Sonntag F., Behrens S., Schmieder F., Voigt B., Meyer M. (2022). Human endothelial cells from an endothelium in freestanding collagen hollow filaments fabricated by direct extrusion printing. Biomater. Biosyst..

[34] Xi Y., Deng X., Shu Z., Yang C. (2024). Probing nanoscale structural response of collagen fibril in human Acilles tendon during loading using in situ SAXS. J. Mechanic. Behav. Biomed. Mater..

[35] Islam M.M.I., Saha A., Trisha F.A., Gonzalez-Andrades M., Patra H.K., Griffith M., Chodosh J., Rajaiya J. (2024). An *in vitro* 3-dimensional collagen-based corneal construct with innervation using human corneal cell lines. Ophthamol. Sci..

[36] Qiao H., Shibaki A., Long H.A., Wang G., Li Q., Nishie W., Abe R., Akiyama M., Shimizu H., McMillan J.R. (2009). Collagen XVII participates in keratinocyte adhesion to collagen IV, and in p38MAPK-dependent migration and cell signaling. J. Investig. Dermatol..

[37] Wang D., Chang F., Guo Z., Chen M., Feng T., Zhang M., Cui X., Jian Y., Li J., Li Y. (2024). The influence of type I and III collagen on the proliferation, migration and differentiation of myoblasts. Tissue Cell.

[38] Wu B., Cheng K., Liu M., Liu J., Jiang D., Ma S., Yan B., Lu Y. (2022). Construction of hyperelastic model of human periodontal ligament based on collagen fibers distribution. J. Mechanic. Behav. Biomed. Mater..

[39] Vignesh V., Kavalappa Y.P., Ponesakki G., Madhan B., Shanmugam G. (2024). Lutein, a carotenoid found in numerous plants and the human eye, demonstrates the capacity to bundle collagen fibrils. Int. J. Biol. Macromol..

[40] Ben C., Liu X., Shen T., Song Y., Li H., Pan B., Hou W., Liu T., Luo P., Ma B. (2021). A recombinant human collagen hydrogel for the treatment of partial-thickness burns: A prospective, self-controlled clinical study. Burns.

[41] Matsuda A., Hasegawa T., Ikeda Y., Wada A., Ikeda S. (2024). Histological and molecular restoration of type VII collagen in recessive dystrophic epidemolysis bullosa mouse skin by topical injection of keratinocyte-like cells differentiated from human adipose-derived mesenchymal stromal cells. J. Dermatol. Sci..

[42] Fischer J., Heidrova A., Hermanova M., Bednarik Z., Joukal M., Bursa J. (2024). Structural parameters defining distribution of collagen fiber directions in human carotid arteries. J. Mechanic. Behav. Biomed. Mater..

[43] Guo Y., Hu Z., Chen J., Zhang Z., Liu Q., Li J., Yang J., Ma Z., Zhao J., Hu J. (2023). Injectable TG-linked recombinant human collagen hydrogel loaded with bFGF for rat cranial defect repair. Int. J. Biol. Macromol..

[44] Wang H., Geng X., AI F., Yu Z., Zhang Y., Zhang B., Lv C., Gao R., Yue B., Dou W. (2024). Nuciferine alleviates collagen-induced arthritic in rats by inhibiting the proliferation and invasion of human arthritis-derived fibroblast-like synoviocytes and rectifying Th17/Treg imbalance. Chin. J. Nat. Med..

[45] Lin S.-N., Musso A., Wang J., Mukherjee P.K., West G.A., Mao R., Lyu R., Li J., Zhao S., Elias M. (2022). Human intestinal myofibroblasts deposited collagen VI enhances adhesiveness for T cells- A novel mechanism for maintenance of intestinal inflammation. Matrix Biol..

[46] Tang X., Liu L., Liu S., Song S., Li H. (2022). MicroRNA-29a inhibits collagen expression and induces apoptosis in human fetal scleral fibroblasts by targeting the Hsp47/Smad3 signaling pathway. Exp. Eye Res..

[47] Kaviani M., Keshtkar S., Sarvestani F.S., Azarpira N., Yaghobi R., Aghdaei M.H., Geramizadeh B., Esfandiari E., Shamsaeefar A., Nikeghbalian S. (2021). The potential of the incorporated collagen microspheres in alginate hydrogel as an engineered three-dimensional microenvironment to attenuate apoptosis in human pancreatic islets. Acta Histochem..

[48] Zamani M., Zahedian A., Tanideh N., Khodabandeh Z., Koohpeyman F., Khazraei H., Zare S., Zarei M., Hosseini S.V. (2023). Comparison effect of collagen/P3HB composite scaffold and human amniotic membrane loaded with mesenchymal stem cells on colon anastomosis healing in male rats. Biochem. Biophys. Res. Commun..

[49] Sun M., Connizzo B.K., Adams S.M., Freedman B.R., Wenstrup R.J., Soslowsky L.J., Birk D.E. (2015). Targeted deletion of collagen V in tendos and ligaments results in a classic Ehlers-Danlos syndrome joint phenotype. Am. J. Pathol..

[50] Sun W., Shahrajabian M.H., Cheng Q. (2019). The insight and survey on medicinal properties and nutritive components of shallot. J. Med. Plants. Res..

[51] Mohabeer A.L., Kroetsch J.T., McFadden M., Khosraviani N., Broekelmann T.J., Hou G., Zhang H., Zhou Y.-Q., Wang M., Gramolini A.O. (2021). Deletion of type VIII collagen reduces blood pressure, increases carotid artery functional distensibility and promotes elastin deposition. Matrix Biol. Plus..

[52] Mohabeer A.L., Hou G., Zhang H., Kroetsch J., Bolz S.S., Heximer S., Assoian R., Bendeck M. (2018). The role of type VIII collagen in arterial vessel stiffening. Atheroscler. Suppl..

[53] Stavusis J., Micule I., Wright N.T., Straub V., Topf A., Oliveira L.P.-D., Dominguez-Gonzalez C., Inashkina I., Kidere D., Chrestian N. (2020). Collagen VI-related limb-girdle syndrome caused by frequent mutation in COL6A3 gene with conflicting reports of pathogencicity. Neuromuscul. Disorder..

[54] Ehnis T., Dieterich W., Bauer M., Lampe B.V., Schuppan D. (1996). A chondroitin/dermatan sulfate form of CD44 is a receptor for collagen XIV (Undulin). Exp. Cell Res..

[55] Wong H.H., Seet S.H., Bascom C.C., Isfort R.J., Bard F.A. (2023). Tonic repression of collagen I by the bradykinin receptor 2 in skin fibroblasts. Matrix Biol..

[56] Taga Y., Kusubata M., Ogawa-Goto K., Hattori S. (2012). Development of a novel method for analyzing collagen *O*-glycoylations by hydrazide chemistry. Mol. Cell. Proteom..

[57] Voziyan P., Bown K.L., Uppuganti S., Leser M., Rose K.L., Nyman J.S. (2024). A map of glycation and glycoxidation sites in collagen I of human cortical bone: Effects of sex and type 2 diabetes. Bone.

[58] Wu Z., Korntner S.H., Mullen A.M., Zeugolis D.I. (2021). Collagen type II: From biosynthesis to advanced biomaterials for cartilage engineering. Biomater. Biosys..

[59] Zhu J., Xu H.-N., Lin T., Xia Z.-J. (2024). Silencing of cysteine and serine rich nuclear protein 1 inhibits apoptosis, senescence and collagen degradation in human-derived vaginal fibroblasts in response to oxidative stress or DNA damage. Exp. Cell Res..

[60] Horiba S., Kawamoto M., Tobita R., Kami R., Ogura Y., Hosoi J. (2023). M1/M2 macrophage skewing is related to reduction in types I, V, and VI collagens with aging in sun-exposed human skin. JID Innovat..

[61] Hou G., Mulholland D., Gronska M.A., Bendeck M.P. (2000). Type VIII collagen stimulates smooth muscle cell migration and matrix metalloproteinase synthesis after arterial injury. Am. J. Pathol..

[62] Zhou X., Ye H., Wang X., Sun J., Tu K., Lv J. (2023). Ursolic acid inhibits human dermal fibroblasts hyperproliferation, migration, and collagen deposition induced by TGF-β via regulation the Smad2/3 pathway. Gene.

[63] Li Q., Zheng Q., He J., Li L., Xie X., Liang H. (2022). Has-miR-142-3p reduces collagen I in human scleral fibroblasts by targeting TGF-β1 in high myopia. Exp. Eye Res..

[64] Marin S., Godet I., Nidadavolu L.S., Tian J., Dickinson L.E., Walston J.D., Gilkes D.M., Abadir P.M. (2022). Valsartan and sacubitril combination treatment enhances collagen production in older adult human skin cells. Exp. Gerontol..

[65] Sun W., Shahrajabian M.H., Cheng Q. (2021). Natural dietary and medicinal plants with anti-obesity therapeutics activities for treatment and prevention of obesity during lock down and in post-Covid-19 eta. Appl. Sci..

[66] Sun W., Shahrajabian M.H., Cheng Q. (2021). Fenugreek cultivation with emphasis on historical aspects and its uses in traditional medicine and modern pharmaceutical science. Mini-Rev. Med. Chem..

[67] Sun W., Shahrajabian M.H. (2023). The application of arbuscular mycorrhizal fungi as microbial biostimulant, sustainable approaches in modern agriculture. Plants.

[68] Sun W., Shahrajabian M.H. (2023). Therapeutic potential of phenolic compounds in medicinal plants-natural health products for human health. Molecules.

[69] Ahmed M., Verma A.K., Malik M.A., Alzahrani K.A., Patel R. (2023). Probing the impact of alkyl chain length of imidazolium ionic liquids on the conformational stability of collagen type-I from skin of *Lutjanus erythropterus*. J. Mol. Struct..

[70] Khalilimofrad Z., Baharifar H., Asefnejad A., Khoshnevisan K. (2023). Collagen type I cross-linked to gelatin/chitosan electrospun mats: Application for skin tissue engineering. Mater. Today Commun..

[71] Sun Y., Cheng Y., Zhao H., Wang J., Liu Y., Bai J., Hu C., Shang Z. (2024). Lactate-driven type I collagen deposition facilitates cancer stem cell-like phenotype of head and neck squamous cell carcinoma. iScience.

[72] Zhang M., Gao T., Han Y., Xue D., Jiang S., Li Q. (2023). Improvement of structural, rheological, and physicochemical properties of type I collagen by calcium lactate combined with ultrasound. Ultrason. Sonochem..

[73] Gao Y., Ma K., Kang Y., Liu W., Liu X., Long X., Hayashi T., Hattori S., Mizuno K., Fujisaki H. (2022). Type I collagen reduces lipid accumulation during adipogenesis of preadipocytes 3T3-L1 via the YAP-mTOR-autophagy axis. Biochim. Biophys. Acta. Mol. Cell Biol. Lipid..

[74] Ghosh D.K., Udupa P., Shrikondawar A.N., Bhavani G.S.L., Shah H., Ranjan A., Girisha K.M. (2023). Mutant MESD links cellular stress to type I collagen aggregation in osteogenesis imperfecta type XX. Matrix Biol..

[75] Preston S.E.J., Bartish M., Richard V.R., Aghigh A., Goncalves C., Smith-Voudouris J., Huang F., Thebault P., Cleret-Buhot A., Lapointe R. (2022). Phosphorylation of eIF4E in the stroma drives the production and spatial organization of collagen type I in the mammary gland. Matrix Biol..

[76] Noohi P., Mahdavi S.S., Abdekhodaie M.J., Nekoofar M.H., Baradaran-Raffi A. (2023). Photoreactive hydrogels based on type I collagen extracted from different sources as scaffolds for tissue engineering applications: A comparative study. Materialia.

[77] Ito A., Yamamoto M., Ikeda K., Sato M., Kawabe Y., Kamihira M. (2015). Effects of type IV collagen on myogenic characteristics of IGF-I gene-engineered myoblasts. J. Biosci. Bioengin..

[78] Wu B., Meng C., Wang H., Jia C., Zhao Y. (2016). Changes of proteoglycan and collagen II of the adjacent intervertebral disc in the cervical instability models. Biomed. Pharmacother..

[79] Chung H.J., Jensen D.A., Gawron K., Steplewski A., Fertala A. (2009). R992C (p.R1192C) substitution in collagen II alters the structure of mutant molecules and induces the unfolded protein response. J. Mol. Biol..

[80] Li W., Kobayashi T., Meng D., Miyamoto B., Tsutsumi N., Ura K., Takagi Y. (2021). Free radical scavenging activity of type II collagen peptides and chondroitin sulfate oligosaccharides from by-products of mottled skate processing. Food Biosci..

[81] Meng D., Tanaka H., Kobayashi T., Hatayama H., Zhang X., Ura K., Yunoki S., Takagi Y. (2019). The effect of alkaline pretreatment on the biochemical characteristics and fibril-forming abilities of types I and II collagen extracted from bester sturgeon by-products. Int. J. Biol. Macromol..

[82] Iqbal M., Waqas M., Mo Q., Shahzad M., Zeng Z., Qamar H., Mehmood K., Kulyar M.F.A., Nawaz S., Li J. (2023). Baicalin inhibits apoptosis and enhances chondrocyte proliferation in thiram-induced tibial dyschondroplasia in chickens by regulating Bcl-2/Caspase-9 and Sox-9/Collagen-II expressions. Ecotoxicol. Environ. Saf..

[83] Caradu C., Brunet C., Spampinato B., Stenson K., Ducasse E., Puges M., Berard X. (2022). Contemporary results with the biosynthetic glutaraldehyde denatured ovine collagen graft (Omniflow II) in lower extremity arterial revascularization in a septic context. Annal. Vasc. Surg..

[84] Tiku M.L., Madhan B. (2016). Preserving the longevity of long-lived type II collagen and its implication for cartilage therapeutics. Age. Res. Rev..

[85] Wu P., Yuan Y., Chen L., Chen M., Chiou B.-S., Liu F., Zhong F. (2023). Effects of gastrointestinal digestion on the cell bioavailability of sodium alginate coated liposomes containing DPP-IV inhibition active collagen peptides. Food Biosci..

[86] Zhu M., Metzen F., Hopkinson M., Betz J., Heilig J., Sodhi J., Imhof T., Niehoff A., Birk D.E., Izu Y. (2023). Ablation of collagen XII disturbs joing extracellular matrix organization and causes patellar subluxation. iScience.

[87] Hua W.-B., Wu X.-H., Zhang Y.-K., Song Y., Tu J., Kang L., Zhao K.-C., Li S., Wang K., Liu W. (2017). Dysregulated miR-127-5p contributes to type II collagen degradation by targeting matrix metalloproteinase-13 in human intervertebral disc degeneration. Biochimie.

[88] Viljanen J., Lonnblom E., Ge C., Yang J., Cheng L., Aldi S., Cai W., Kastbom A., Sjowall C., Gjertsson I. (2020). Synthesis of an array of triple-helical peptides from type II collagen for multiplex analysis of autoantibodies in rheumatoid arthritis. ACS Chem. Biol..

[89] Ugurlu E., Duysak O., Kardas G., Sayin S., Saygili E.I., Dogan S. (2023). Alginate modified collagen for rapid, durable and effective biosorption of Pb(II) ions from an aqueous solution. Regional Stud. Marine Sci..

[90] Vazquez-Portalatin N., Kilmer C.E., Panitch A., Liu J.C. (2016). Characterization of collagen type I and II blended hydrogels for articular cartilage tissue engineering. Biomacromolecules.

[91] Zhao Y., Lu K., Piao X., Song Y., Wang L., Zhou R., Gao P., Khong H.Y. (2023). Collagens for surimi gel fortification: Type-dependent effects and the difference between type I and type II. Food Chem..

[92] Hu X., Jin M., Sun K., Zhang Z., Wu Z., Shi J., Liu P., Yao H., Wang D.-A. (2024). Type II collagen scaffolds repair critical-sized osteochondral defects under induced conditions of osteoarthritis in rat knee joints via inhibiting TGF-B-Smad1/5/8 signaling pathway. Bioact. Mater..

[93] Nham G.T.H., Zhang X., Asou Y., Shinomura T. (2019). Expression of type II collagen and aggrecan genes is regulated through distinct epigenetic modifications of their multiple enhancer elements. Gene.

[94] An B., Abbonante V., Yigit S., Balduini A., Kaplan D.L., Brodsky B. (2014). Definition of the native and denatured type II collagen binding site for fibronectin using a recombinant collagen system. J. Biol. Chem..

[95] Chang D.P., Guilak F., Jay G.D., Zauscher S. (2014). Interaction of lubricin with type II collagen surfaces: Adsorption, friction, and normal forces. J. Biomech..

[96] Yao L., Flynn N. (2018). Dental pulp stem cell-derived chondrogenic cells demonstrate differential cell motility in type I and type II collagen hydrogels. Spine J..

[97] Santiago S., Enwereji N., Jiang C., Durrani K., Chaudhry S., Lu J. (2024). Ocular and eyelid involvement in collagen vascular diseases. Part II. Dermatomyositis, scleroderma, and sarcoidosis. Clin. Dermatol..

[98] Arita M., Fertala J., Hou C., Steplewski A., Fertala A. (2015). Mechanisms of aberrant organization of growth plates in conditional transgenic mouse model of spondyloepiphyseal dysplasia associated with the R992C substitution in collagen II. Am. J. Pathol..

[99] Greene C.A., Green C.R., Dickinson M.E., Johnson V., Sherwin T. (2016). Keratocytes are induced to produce collagen type II: A new strategy for in vivo corneal matrix regeneration. Exp. Cell Res..

[100] Li Y., Tang J., Hu Y. (2014). Dimethyl fumarate protection against collagen II degradation. Biochem. Biophys. Res. Commun..

[101] Wang W., Ji Y., Yang W., Zhang C., Angwa L., Jin B., Liu J., Lv M., Ma W., Yang J. (2020). Inhibitors of apoptosis proteins (IAPs) are associated with T-2 toxin-induced decreased collagen II in mouse chondrocytes in vitro. Toxicon.

[102] Fertala A. (2020). Three decades of research on recombinant collagens: Reinventing the wheel or developing new biomedical products?. Bioengineering.

[103] Groen S.S., Sinkeviciute D., Bay-Jensen A.-C., Thudium C.S., Karsdal M.A., Thomsen S.F., Lindemann S., Wekmann D., Bliar J., Staunstrup L.M. (2021). A serological type II collagen neoepitope biomarker reflects cartilage breakdown in patients with osteoarthritis. Osteoarthr. Cartil. Open.

[104] Xu R., Zheng L., Su G., Luo D., Lai C., Zhao M. (2021). Protein solubility, secondary structure and microstructure changes in two types of undenatured type II collagen under different gastrointestinal digestion conditions. Food Chem..

[105] Fakatava N., Mitarai H., Yuda A., Haraguchi A., Wada H., Hasegawa D., Maeda H., Wada N. (2023). Actin alpha 2, smooth muscle, a transforming growth factor-β1-induced factor, regulates collagen production in human periodontal ligament cells via Smad2/3 pathway. J. Dent. Sci..

[106] Barber L.A., Abbott C., Nakhate V., Do A.N.D., Blissett A.R., Marini J.C. (2019). Longitudinal growth curves for children with classical osteogenesis imperfecta (types III and IV) caused by structural pathogenic variants in type I collagen. Genet. Med..

[107] Guiliani A.M., Frederiksen P., Skovgaard E., Lonsmann I., Karsdal M., Bendtsen F., Pedersen J.S., Trebicka J., Leeming D. (2024). THU-243 macrophage driven fibrosis resolution assessed by a cross-linked and MMP degraded type III collagen fragment (CTX-III) declines with age and is prognostic for survival in chronic liver disease. J. Hepatol..

[108] Yang L., Wu H., Lu L., He Q., Xi B., Yu H., Luo R., Wang Y., Zhang X. (2021). A tailored extracellular matrix (ECM)-Mimetic coating for cardiovascular stents by stepwise assembly of hyaluronic acid and recombinant human type III collagen. Biomaterials.

[109] Dong S., Wang H., Ji H., Hu Y., Zhao S., Yan B., Wang G., Lin Z., Zhu W., Lu J. (2023). Development and validation of a collagen signature to predict the prognosis of patients with stage II/III colorectal cancer. iScience.

[110] Jia Y., Han Y., Zhang Y., Li L., Zhang B., Yan X. (2024). Multifunctional type III recombinant human collagen incorporated sodium alginate hydrogel with sustained release of extra cellular vehicles for wound healing multimodal therapy in diabetic mice. Regenerative Ther..

[111] Hsueh C.-M., Tseng T.-Y., Lin H.-M., Lee S.-L., Huang Y.-D., Dong C.-Y. (2018). Optical discrimination of type I and type III collagen through second order susceptibility imaging. Optik.

[112] Li H., You S., Yang X., Liu S., Hu L. (2022). Injectable recombinant human collagen-derived material with high cell adhesion activity limits adverse remodelling and improves pelvic floor function in pelvic floor dysfunction rats. Biomater. Adv..

[113] Fang J., Ma Z., Liu D., Wang Z., Cheng S., Zheng S., Wu H., Xia P., Chen X., Yang R. (2023). Co-expression of recombinant human collagen α1(III) chain with viral prolyl 4-hydroxylase in *Pichia pastoris* GS115. Protein Express. Purif..

[114] Yang Y., Ritchie A.C., Everitt N.M. (2021). Using type III recombinant human collagen to construct a series of highly porous scaffolds for tissue regeneration. Colloids Surf. Bioninter..

[115] Uchida Y., Shimoyama E., Hiruta N., Tabata T. (2021). Detection of early stage of human coronary atherosclerosis by angioscopic imaging of collagen subtypes. J. Cardiol..

[116] Sofii I., Dipoyono W., Prima H., Sari Y.M., Fauzi A.R., Gunadi (2020). The effect of different suturing materials for abdominal fascia wound closure on the collagen I/III expression ration in rats. Annal Med. Surg..

[117] Fu D., Xiang Y., Xiang Z., Zhang J., Yang F., Yang L., Zhao J., Wang Y. (2024). Optimizing small-diameter vascular grafts: Harnessing the power of recombinant human type III collagen (rhCOLIII) for enhanced antiplatelet and endothelialization performance. Chem. Eng. J..

[118] Genovese F., Goncalves I., Nielsen S.H., Karsdal M.A., Edsfeldt A., Nilsson J., Shore A.C., Natali A., Khan F., Shami A. (2024). Plasma levels of PRO-C3, a type III collagen synthesis marker, are associated with arterial stiffness and increased risk of cardiovascular death. Atherosclerosis.

[119] Pires V., Pecher J., Nascimento S.D., Maurice P., Bonnefoy A., Saddoniville A., Amant C., Fauvel-Lafeve F., Legrand C., Rochette J. (2007). Type III collagen mimetic peptides designed with anti- or pro-aggregant activities on human platelets. Eur. J. Med. Chem..

[120] Ge Y., Guo G., Liu K., Yang F., Yang L., Wang Y., Zhang X. (2022). A strategy of functional crosslinking acellular matrix in blood-contacting implantable devices with recombinant humanized collagen type III (rhCOLIII). Compos. Part B Eng..

[121] Boudako S.P., Engel J. (2004). Structure formation in the C terminus of type III collagen guides disulfide cross-linking. J. Mol. Biol..

[122] Shuai Q., Liang Y., Xu X., Halbiyat Z., Wang X., Cheng J., Liu J., Huang T., Peng Z., Wang L. (2023). Sodium alginate hydrogel integrated with type III collagen and mesenchymal stem cell to promote endometrium regeneration and fertility restoration. Int. J. Biol. Macromol..

[123] Li C.-H., Chen W., Hu J.-Q., Wang Y.-D., Wang S.-D., Zeng Y.-Q., Wang H. (2016). miRNA-29a targets *COL3A1* to regulate the level of type III collagen in pig. Gene.

[124] Adams D.H., Shou Q., Wohlmuth H., Cowin A.J. (2016). Native Australian plant extracts differentially induce collagen I and collagen III in vitro and could be important targets for the development of new wound healing therapies. Fitoterapia.

[125] Kuivaniemi H., Tromp G. (2019). Type III collagen (COL3A1): Gene and protein structure, tissue distribution, and associated diseases. Gene.

[126] Makuszewska M., Bonda T., Cieslinska M., Bialuk I., Winnicka M.M., Niemczyk K. (2020). Expression of collagen type III in healing tympanic membrane. Int. J. Pediatr. Otorhinolaryngol..

[127] Weerakoon A.T., Condon N., Cox T.R., Sexton C., Cooper C., Meyers I.A., Thomson D., Ford P.J., Roy S., Symons A.L. (2022). Dynamic dentin: A quantitative microscopic assessment of age and spatial changes to matrix architecture, peritubular dentin, and collagens types I and III. J. Struct. Biol..

[128] Wang C., Brisson B.K., Terajima M., Li Q., Hoxha K.H., Han B., Goldberg A.M., Liu X.S., Marcolongo M.S., Enomoto-Iwamoto M. (2020). Type III collagen is a key regulator of the collagen fibrillar structure and biomechanics of articular cartilage and meniscus. Matrix Biol..

[129] Liu T., Qiu C., Lu H., Li H., Zhu S., Ma L. (2023). A novel recombinant human collagen hydrogel as minced split-thickness skin graft overlay to promote full-thickness skin defect reconstruction. Burns.

[130] Pinheiro L.C.L., Pupim A.C.E., Pereira E.R., Ahrens T.M., Mendonca A.C., Francelino A.L., Araujo E.J.D.A., Guembarovski A.F.M.L., Fuganti P.E., Vanzela A.L.L. (2024). Deposition of collagen III and alterations in basement membrane integrity as candidate prognostic markers in prostate cancer. Exp. Cell Res..

[131] Mou S., Wang Q., Shi B., Gu L., Ni Z. (2009). Hepatocyte growth factor suppresses transforming growth factor-Beta-1 and type III collagen in human primary renal fibroblasts. Kaohsiung J. Med. Sci..

[132] Nurmenniemi S., Koivula M.-K., Nyberg P., Tervahartiala T., Sorsa T., Mattila P.S., Salo T., Risteli J. (2012). Type I and III collagen degradation products in serum predict patient survival in head and neck squamous cell carcinoma. Oral Oncol..

[133] Jaleel G.A.A., Saleh D.O., Al-Awdan S.W., Hassan A., Asaad G.F. (2020). Impact of type III collagen on monosodium iodoacetate-induced osteoarthritis in rats. Heliyon.

[134] Abbonante V., Gruppi C., Battiston M., Zulian A., Buduo C.A.D., Chrisam M., Sereni L., Laurent P.-A., Semplicini C., Lombardi E. (2021). Ablation of collagen VI leads to the release of platelets with altered function. Blood Adv..

[135] Jaikumar D., Baskaran B., Vaidyanathan V.G. (2017). Effect of chromium (III) gallate complex on stabilization of collagen. Int. J. Biol. Macromol..

[136] Sato K., Yomogida K., Wada T., Yorihuzi T., Nishimune Y., Hosokawa N., Nagata K. (2002). Type XXVI collagen, a new member of the collagen family, is specifically expressed in the testis and ovary. J. Biol. Chem..

[137] Balancin M.L., Teodoro W.R., Baldavira C.M., Prieto T.G., Farhat C., Velosa A.P., Souza P.D.C., Yaegashi L.B., AbSaber A.M., Takagaki T.Y. (2020). Different histological patterns of type-V collagen levels confer a matrices-privileged tissue microenvironment for invasion in malignant tumors with prognostic value. Pathol. Res. Pract..

[138] Paladin L., Tosatto S.C.E., Minervini G. (2015). Structure in silico dissection of the collagen V interactome to identify genotype-phenotype correlations in classic Ehlers-Danlos Syndrome (EDS). FEBS Lett..

[139] Martins V., Silva A.L.D., Teodoro W.R., Velosa A.P.P., Balancin M.L., Cruz F.F., Silva P.L., Rocco P.R.M., Capelozzi V.L. (2020). *In situ* evidence of collagen V and signaling pathway of found inflammatory zone 1 (FIZZ1) is associated with silicotic granuloma in lung mice. Pathol. Res. Pract..

[140] Sun M., Luo E.Y., Adams S.M., Adams T., Ye Y., Shetye S.S., Soslowsky L.J., Birk D.E. (2020). Collagen XI regulates the acquisition of collagen fibril structure, organization and functional properties in tendon. Matirx Biol..

[141] Kahai S., Vary C.P.H., Gao Y., Seth A. (2004). Collagen, type V, alpha1 (COL5A1) is regulated by TGF-beta in osteoblasts. Matrix Biol..

[142] Smith G.N., Williams J.M., Brandt K.D. (1987). Effect of polyanions on fibrillogenesis by type XI collagen. Collagen Relat. Res..

[143] Yokota T., McCourt J., Ma F., Ren S., Li S., Kim T.-H., Kurmangaliyev Y.Z., Nasiri T., Ahadian S., Nguyen T. (2020). Type V collagen in scar tissue regulates the size of scar after heart injury. Cell.

[144] Kostin S., Richter M., Ganceva N., Sasko B., Giannakopoulos T., Ritter O., Szalay Z., Pagonas N. (2024). Atrial fibrillation in human patients is associated with increased collagen type V and TGFbeta1. IJC Heart Vasc..

[145] Moradi-Ameli M., Chassey B.D., Farjanel J., Rest M.V.D. (1998). Different splice variants of cartilage α1(X1) collagen chain undergo uniform amino-terminal processing. Matrix Biol..

[146] Berendsen A.D., Bronckers A.L.J.J., Smit T.H., Walboomers X.F., Everts V. (2006). Collagen type V enhances matrix contraction by human periodontal ligament fibroblasts seeded in three-dimensional collagen gels. Matrix Biol..

[147] Bi H., Ye K., Jin S. (2020). Proteomic analysis of decellularized pancreatic matrix identifies collagen V as a critical regulator for islet organogenesis from human pluripotent stem cells. Biomaterials.

[148] Zaffiri L., Shah R.J., Stearman R.S., Rothhaar K., Emtiazjoo A.M., Yoshimoto M., Fisher A.J., Mickler E.A., Gartenhaus M.D., Cohort L.T.O.G. (2019). Collagen type-V is a danger signal associated with primary graft dysfunction in lung transplantation. Transpl. Immunol..

[149] Breuls R.G.M., Klumpers D.D., Everts V., Smit T.H. (2009). Collagen type V modulates fibroblast behavior dependent on substrate stiffness. Biochem. Biophys. Res. Commun..

[150] Braun R.K., Martin A., Shah S., Iwashima M., Medina M., Byrne K., Sethupathi P., Wigfield C.H., Brand D.D., Love R.B. (2010). Inhibition of bleomycin-induced pulmonary fibrosis through pre-treatment with collagen type V. J. Heart Lung Transplant..

[151] McLeod O., Duner P., Samnegard A., Tornvall P., Nilsson J., Hamsten A., Bengtsson E. (2015). Autoantibodies against basement membrane collagen type IV are associated with myocardial infarction. IJC Heart Vasc..

[152] Bordini M., Mazzoni M., Nunzio M.D., Zappaterra M., Sirri F., Meluzzi A., Petracci M., Soglia F. (2024). Time course evaluation of collagen type IV in *Pectoralis major* muscles of broiler chickens selected for different growth-rates. Poultry Sci..

[153] Duncan M., Kalluri R. (2011). Type XVIII collagen induced signaling via α1β1 integrin in hepatocytes is essential for surviving liver injury. Gastroenterology.

[154] Anazo C., Lopez-Jimenez A.J., Rafi M., Vega-Montoto L., Zhang M.-Z., Hudson B., Vanacore R.M. (2016). Lysyl oxidase-like-2 cross-links collagen IV of glomerular basement membrane. J. Biol. Chem..

[155] Komarowska M., Szymanska B., Oldak L., Sankiewicz A., Matuszczak E., Gorodkiewicz E., Debek W., Milewski R., Hermanowicz A. (2020). Plasma level of laminin 5 and collagen IV in cryptorchidism. Adv. Med. Sci..

[156] Khan T., Muise E.S., Iyengar P., Wang Z.V., Chandalia M., Abate N., Zhang B.B., Bonaldo P., Chua S., Scherer P.E. (2009). Metabolic dysregulation and adipose tissue fibrosis: Role of collagen VI. Mol. Cell. Biol..

[157] Chen C.-H., Yeh M.-L., Geyer M., Wang G.-J., Huang M.-H., Heggeness M.H., Hook M., Luo Z.-P. (2006). Interactions between collagen IX and biglycan measured by atomic force microscopy. Biochem. Biophys. Res. Commun..

[158] Wegener H., Leineweber S., Seeger K. (2013). The vWFA2 domain of type VII collagen is responsible for collagen binding. Biochem. Biophys. Res. Commun..

[159] Lopes J., Adiguzel E., Gu S., Liu S.-L., Hou G., Heximer S., Assoian R.K., Bendeck M.P. (2013). Type VIII collagen mediates vessel wall remodeling after arterial injury and fibrous cap formation in atherosclerosis. Am. J. Pathol..

[160] Lorenzo-Gomez R., Miranda-Castro R., Toyos J.R.D.I., Alvarez N.D.I.S., Castanon M.J.L. (2022). Aptamers targeting a tumor-associated extracellular matrix component: The human mature collagen Xiα1. Anal. Chim. Acta..

[161] Snellman A., Tu H., Vaisanen T., Kvist A.-P., Huhtala P., Pihlajaniemi T. (2000). A short sequence in the N-terminal region is required for the trimerization of type XIII collagen and is conserved in other collagenous transmembrane proteins. EMBO J..

[162] Woods A., James C., Underhill T.M., Beier F., CIHR Group in Skeletal Development and Remodeling (2004). Identification of the putative collagen X gene from the pufferfish *Fugu rubripes*. Gene.

[163] Oxford J.T., Doege K.J., Morris N.P. (1995). Alternative exon splicing withing the amino-terminal nontriple-helical domain the rat pro-a1(XI) collagen chain generates multiple forms of the mRNA transcript which exhibit tissue-dependent variation. J. Biol. Chem..

[164] Zhidkova N.I., Justice S.K., Mayne R. (1995). Alternative mRNA processing occurs in the variable region of the pro-α1(XI) and pro-α2(XI) collagen chains. J. Biol. Chem..

[165] Fichard A., Kleman J.-P., Ruggiero F. (1995). Another loot at collagen V and XI molecules. Matrix Biol..

[166] Rodriguez R.R., Seegmiller R.E., Stark M.R., Bridgewater L.C. (2004). A type XI collagen mutation leads to increased degradation of type II collagen in articular cartilage. Osteoarthr. Cartil..

[167] Douglas S.P., Jenkins J.M., Kadler K.E. (1998). Collagen IX: Evidence for a structural association between NC4 domains in cartilage and a novel cleavage site in the α1(IX) chain. Matrix Biol..

[168] Moroi M., Induruwa I., Farndale R.W., Jung S.M. (2022). Factor XIII is a new widely identified binding partner for platelet collagen receptor GPVI-dimer- An interaction that may modulate fibrin crosslinking. Res. Pract. Thromb. Haemost..

[169] Ichimura S., Wu J.-J., Eyre D.R. (2000). Two-dimensional peptide mapping of cross-linked type IX collagen in human cartilage. Arch. Biochem. Biophys..

[170] Ehnis T., Dieterich W., Bauer M., Kresse H., Schuppan D. (1997). Localization of a binding site for the proteoglycan decorin on collagen XIV (Undulin). J. Biol. Chem..

[171] Chai C.-J., Sun Y., Chi R.-F., Yang H.-Y., Yang B., Li B. (2024). Astragaloside IV alleviates LPS-induced cardiomyocyte hypertrophy and collagen expression associated with CCL2-mediated activation of NF-κB signaling pathway. Biochem. Biophys. Res. Commun..

[172] Soder S., Poschl E. (2004). The NC1 domain of human collagen IV is necessary to initiate triple helix formation. Biochem. Biophys. Res. Commun..

[173] Boudko S.P., Engel J., Okuyama K., Mizuno K., Bachinger H.P., Schumacher M.A. (2008). Crystal structure of human type III collagen Gly991-Gly1032 cystine knot-containing peptide shows both 7/2 and 10/3 triple helical symmetries. J. Biol. Chem..

[174] Roy A., Alnakhli T.H., Gauld J.W. (2022). Computational insights into the formation and nature of the sulfilimine bond in collagen IV. RSC Adv..

[175] Wilson R., Freddi S., Chan D., Cheah K.S.E., Bateman J.F. (2005). Misfolding of collagen X chains harboring Schmid metaphyseal chondrodysplasia mutations results in aberrant disulfide bond formation, intracellular retention, and activation of the unfolded protein response. J. Biol. Chem..

[176] Wu J.-J., Weis M.A., Kim L.S., Carter B.G., Eyre D.R. (2009). Differences in chain usage and cross-linking specificities of cartilage type V/XI collagen isoforms with age and tissue. J. Biol. Chem..

[177] Peng Q., Chen J., Wang R., Zhu H., Han C., Ji X., Pan Y. (2022). The sex determination gene *doublesex* regulates expression and secretion of the basement membrane protein collagen IV. J. Genet. Genom..

[178] Ito K., Shinomura T. (2016). Development and application of a new silent reporter system to quantitate he activity of enhancer elements in the type II collagen gene. Gene.

[179] Ferreira L.R., Norris K., Smith T., Hebert C., Sauk J.J. (1996). Hsp47 and other ER-resident molecular chaperones form heterocomplexes with each other and with collagen type IV chains. Connect. Tissue Res..

[180] Su C., Su B., Tang L., Zhao Y., Zhou C. (2007). Effects of collagen IV on cisplatin-induced apoptosis of non-small cell lung cancer cells. Cancer Investig..

[181] Iida M., Yamamoto M., Ishiguro Y.S., Yamazaki M., Ueda N., Honjo H., Kamiya K. (2014). Urinary type IV collagen is related to left ventricular diastolic function and brain natriuretic peptide in hypertensive patients with prediabetes. J. Diabetes Complicat..

[182] Liang X., Osman T.A.-H., Sapkota D., Neppelberg E., Lybak S., Liavaag P.G., Johannessen A.C., Jacobsen H.K., Enger P.O., Costea D.E. (2014). Rapid adherence to collagen IV enriches for tumor initiating cells in oral cancer. Eur. J. Cancer.

[183] Chen V.M., Shelke R., Nystrom A., Laver N., Sampson J.F., Zhiyi C., Bhat N., Panjwani N. (2018). Collagen VII deficient mice show morphologic and histologic corneal changes that phenotypically mimic human dystrophic epidermolysis bullosa of the eye. Exp. Eye Res..

[184] Pastor-Pareja J.C., Xu T. (2011). Shaping cells and organs in *Drosophila* by opposing roles of fat body-secreted collagen IV and perlecan. Dev. Cell.

[185] Ito Y., Iwashita J., Murata J. (2019). Type IV collagen reduces mucin 5AC secretion in three-dimensional cultures human primary airway epithelial cells. Biochem. Biophys. Rep..

[186] Stefano J.T., Guedes L.V., Souza A.A.A.D., Vanni D.S., Alves V.A.F., Carrilho F.J., Largura A., Arrese M., Oliveira C.P. (2021). Usefulness of collagen type IV in the detection of significant liver fibrosis in nonalcoholic fatty liver disease. Ann. Hepatol..

[187] Bai X., Dilworth D.J., Weng Y.-C., Gould D.B. (2009). Developmental distribution of collagen IV isoforms and relevance to ocular diseases. Matrix Biol..

[188] Harumiya S., Gibson M.A., Koshihara Y. (2002). Antisense suppression of collagen VI synthesis results in reduced expression of collagen I in normal human osteoblast-like cells. Biosci. Biotechnol. Biochem..

[189] Akagi A., Tajima S., Nagai Y., Ishibashi A., Yamaguchi N. (1999). Expression of type XVI collagen in human skin fibroblasts: Enhanced expression in fibrotic skin diseases. J. Investig. Dermatol..

[190] Grumati P., Coletto L., Schiavinato A., Castagnaro S., Bertaggia E., Sandri M., Bonaldo P. (2011). Physical exercise stimulates autophagy in normal skeletal muscles but is detrimental for collagen VI-deficient muscles. Autophagy.

[191] Bolduc V., Sizov K., Brull A., Esposito E., Chen G.S., Uapinyoying P., Sarathy A., Johnson K.R., Bonnemann C.G. (2024). Allele-specific CRISPR-Cas9 editing inactivates a single nucleotide variant associated with collagen VI muscular dystrophy. Mol. Ther. Nuc. Acids..

[192] Freire J., Dominguez-Hormaetxe S., Pereda S., Juan A.D., Vega A., Simon L., Gomez-Roman J. (2014). Collagen, type XI, alpha 1: An accurate marker for differential diagnosis of breast carcinoma invasiveness in core needle biopsies. Pathol. Res. Pract..

[193] Ramos-Moreno T., Cifra A., Litsa N.L., Melin E., Ahl M., Christiansen S.H., Gotzsche C.R., Cescon M., Bonaldo P., Loo K.V. (2024). Collagen VI: Role in synaptic transmission and seizure-related excitability. Exp. Neurol..

[194] Pruitt H.C., Guan Y., Liu H., Carey A.E., Brennen W.N., Lu J., Joshu C., Weeraratna A., Lotan T.L., Eisinger-Mathason T.S.K. (2023). Collagen VI deposition mediates stromal T cell trapping through inhibition of T cell motility in the prostate tumor microenvironment. Matrix Biol..

[195] Liu Y., Shimizu H., Hashimoto T. (2003). Immunofluorscence studies using skin sections of recessive dystrophic epidermolysis bullosa patients indicated that the antigen of anti-p200 pemphigoid is not a fragment of type VII collagen. J. Dermatol. Sci..

[196] Bornert O., Kocher T., Gretzmeier C., Liemberger B., Hainzl S., Koller U., Nystrom A. (2019). Generation of rabbit polyclonal human and murine collagen VII monospecific antibodies: A useful tool for dystrophic epidermolysis bullosa therapy studies. Matrix Biol. Plus..

[197] Kruppa D., Peters F., Bornert O., Maler M.D., Martin S.F., Becker-Pauly C., Nystrom A. (2021). Distinct contributions of meprins to skin regeneration after injury- Meprin α a physiological processor of pro-collagen VII. Matrix Biol. Plus.

[198] Udupa P., Shrikondawar A.N., Nayak S.S., Shah H., Ranjan A., Girisha K.M., Bhavani G.S., Ghosh D.K. (2023). Deep intronic mutation in *CRTAP* results in unstable isoforms of the protein to induce type I collagen aggregation in a lethal type of osteogenesis imperfecta type VII. Biochim. Biophys. Acta. Mol. Basis. Dis..

[199] Uzawa K., Yeowell H.N., Yamamoto K., Mochida Y., Tanzawa H., Yamauchi M. (2003). Lysine hydroxylation of collagen in a fibroblast cell culture system. Biochem. Biophys. Res..

[200] Chen M., Keene D.R., Costa F.K., Tahk S.H., Woodley D.T. (2001). The carboxyl terminus of type VII collagen mediates antiparallel dimer formation and constitutes a new antigenic epitope for epidermolysis bullosa acquisita autoantibodies. J. Biol. Chem..

[201] Gretzmeier C., Pin D., Kern J.S., Chen M., Woodley D.T., Bruckner-Tuderman L., Souza M.P.D., Nystrom A. (2022). Systemic collagen VII replacement therapy for advanced recessive dystrophic epidermolysis bullosa. J. Investig. Dermatol..

[202] Mcguire J.D., Walker M.P., Mousa A., Wang Y., Gorski J.P. (2014). Type VII collagen is enriched in the enamel organic matrix associated with the dentin-enamel junction of mature human teeth. Bone.

[203] Woodley D., Hou Y., Tang X., Tan C., Zhang K., Bainvoll L., Li W., Chen M. (2023). Topical type VII collagen increased elastic fiber formation, accelerated wound closure and reduced scarring of diabetic pigskin wounds. J. Investig. Dermatol..

[204] Mann K., Jander R., Korsching E., Kuhn K., Rauterberg J. (1990). The primary structure of a triple-helical domain of collagen type VIII from bovine Descemet’s membrane. FEBS Lett..

[205] Darvish D.M. (2022). Collagen fibril formation *in vitro*: From origin to opportunities. Mater. Today Bio..

[206] Greenhill N.S., Ruger B.M., Hasan Q., Davis P.F. (2000). The a1(VIII) and a2(VIII) collagen chains form two distinct homotrimeric proteins in vivo. Matrix Biol..

[207] Hu X., Dai Z., Pan R., Zhang Y., Liu L., Wang Y., Chen X., Yao D., Hong M., Liu C. (2022). Long-term transplantation human menstrual blood mesenchymal stem cell loaded collagen scaffolds repair endometrium histological injury. Reprod. Toxicol..

[208] Kivirikko S., Heinamaki P., Rehn M., Honkanen N., Myers J.C., Pihlajaniemi T. (1994). Primary structure of the alpha 1 chain of human type XV collagen and exon-intron organization in the 3^/^ region of the corresponding gene. J. Biol. Chem..

[209] Momota R., Naito I., Ninomiya Y., Ohtsuka A. (2011). *Drosophila* type XV/XVIII collagen, Mp, is involved in Wingless distribution. Matrix Biol..

[210] Hansen N.U.B., Willumsen N., Sand J.M.B., Larsen L., Karsdal M.A., Leeming D.J. (2016). Type VIII collagen is elevated in diseases associated with angiogenesis and vascular remodeling. Clin. Biochem..

[211] Grassel S., Bauer R.J. (2013). Collagen XVI in health and disease. Matrix Biol..

[212] Hagg P.M., Horelli-Kuitunen N., Eklund L., Palotie A., Pihlajaniemi T. (1997). Cloning of mouse type XV collagen sequences and mapping of the corresponding gene to 4B1-3. Genomics.

[213] Hagg P.M., Muona A., Lietard J., Kivirikko S., Pihlajaniemi T. (1998). Complete Exon-intron organization of the human gene for the α1 chain of type XV collagen (*COL15A1*) and comparison with the homologous *Col18α1* gene. J. Biol. Chem..

[214] Alexdottir M.S., Bourgonje A.R., Karsdal M.A., Bay-Jensen A.-C., Pehrsson M., Loveikyte R., Van Dullemen H.M., Visschedijk M.C., Festen E.A., Weersma R.K. (2022). Mo1524: Serological biomarkers of type VI and XXII collagen formation predict and monitor infliximab treatment response in patients with Crohn’s disease. Gastroenterology.

[215] Amenta P.S., Briggs K., Xu K., Gamboa E., Jukkola A.F., Li D., Myers J.C. (2000). Type XV collagen in human colonic adenocarcinomas has a different distribution than other basement membrane zone proteins. Hum. Pathol..

[216] Bachinger H.P., Mizuno K., Vranka J.A., Boudko S.P. (2010). Collagen formation and structure. Compr. Nat. Prod. II.

[217] Heljasvaara R., Nyberg P., Luostarinen J., Parikka M., Heikkila P., Rehn M., Sorsa T., Salo T., Pihlajaniemi T. (2005). Generation of biologically active endostatin fragments from human collagen XVIII by distinct matrix metalloproteases. Exp. Cell Res..

[218] Fang Z., Lu X., Du W., Wang X., Yang H., Shi M., Liu T., Xie Y., Wang S., Xu X. (2023). Injectable self-assembled dual-crosslinked alginate/recombinant collagen-based hydrogel for endometrium regeneration. Int. J. Biol. Macromol..

[219] Mor N., Sumiyoshi H., Lee S.Y., Henderson S., Tanaka S., Yoshioka H., Ramirez F., Rattan S., Mor N. (2003). Role of collagen XIX in the nitrergic inhibitory neurotransmission in the lower esophageal sphincter (LES). Gastroenterology.

[220] Jendricke P., Centner C., Zdzieblik D., Gollhofer A., Konig D. (2019). Specific collagen peptides in combination with resistance training improve body composition and regional muscle strength in premenopausal women: A randomized controlled trial. Nutrients.

[221] Li H.-C., Huang C.-C., Chen S.-F., Chou M.-Y. (2005). Assembly of homotrimeric type XXI minicollagen by co-expression of prolyl 4-hydroxylase in stably transfected *Drosophila melanogaster* S2 cells. Biochem. Biophys. Res. Commun..

[222] Delbaere S., Dhooge T., Syx D., Petit F., Goemans N., Destree A., Vanakker O., Rycke R.D., Symoens S., Malfait F. (2020). Novel defects in collagen XII and VI expand the mixed myopathy/Ehlers-Danlos syndrome spectrum and lead to variant-specific alterations in the extracellular matrix. Genet. Med..

[223] Kobayashi T., Kronenberg H. (2005). Minireview: Transcriptional regulation in development of bone. Endocrinology.

[224] An B., Kaplan D.L., Brodsky B. (2014). Engineered recombinant bacterial collagen as an alternative collagen-based biomaterial for tissue engineering. Front. Chem..

[225] Tvaroska I. (2024). Glycosylation modulates the structure and functions of collagen: A review. Molecules.

[226] Arita M., Fertala J., Hou C., Kostas J., Steplewski A., Fertala A. (2017). Prospects and limitations of improving skeletal growth in a mouse model of spondyloepiphyseal dysplasia caused by R992C (p.R1192C) substitution in collagen II. PLoS ONE.

[227] Asamura K., Abe S., Imamura Y., Aszodi A., Suzuki N., Hashimoto S., Takumi Y., Hayashi T., Fassler R., Nakamura Y. (2005). Type IX collagen is crucial for normal hearing. Neuroscience.

[228] Bader H.L., Lambert E., Guiraud A., Malbouyres M., Driever W., Koch M., Ruggiero F. (2013). Zebrafish collagen XIV is transiently expressed in epithelia and is required for proper function of certain basement membranes. J. Biol. Chem..

[229] Carre A.L., James A., Macleod L., Kawai K., Longaker M.T., Lorenz H.P. (2009). High expression of collagen XV and nonfibrillar elastic fibers in embryonic as compared with postnatal skin. J. Am. Coll. Surgen..

[230] Li D., Clark C.C., Myers J.C. (2000). Basement membrane zone type XV collagen is a disulfide-bonded chondroitin sulfate proteoglycan in human tissues and cultures cells. J. Biol. Chem..

[231] Franzke C.-W., Bruckner-Tuderman L., Blobel C.P. (2009). Shedding of collagen XVII/BP180 in skin depends on both ADAM10 and ADAM9. J. Biol. Chem..

[232] Freise C., Bobb V., Querfeld U. (2017). Collagen XIV and a related recombinant fragment protect human vascular smooth muscle cells from calcium-/phosphate-induced osteochondrocytic transdifferentiation. Exp. Cell Res..

[233] Duran I., Csukasi F., Taylor S.P., Krakow D., Becerra J., Bombarely A., Mari-Beffa M. (2015). Collagen duplicate genes of bone and cartilage participate during regeneration of zebradish fin skeleton. Gene Expr. Patt..

[234] Elamaa H., Snellman A., Rehn M., Autio-Harmainen H., Pihlajaniemi T. (2003). Characterization of the human type XVIII collagen gene and proteolytic processing and tissue location of the variant containing a frizzled motif. Matrix Biol..

[235] Arai K., Kasashima Y., Kobayashi A., Kuwano A., Yoshihara T. (2002). TGF-β alters collagen XII and XIV mRNA levels in cultures equine tenocytes. Matrix Biol..

[236] Cabral W.A., Fratzl-Zelman N., Weis M.A., Perosky J.E., Alimasa V., Harris R., Kang H., Makareeva E., Barnes A.M., Roschger P. (2020). Substitution of murine type I collagen A1 3-hydroxylation site alters matrix structure but does not recapitulate osteogenesis imperfecta bone dysplasia. Matrix Biol..

[237] Campbell M.R., Gress C.J., Appleman E.H., Jacenko O. (2004). Chicken collagen X regulatory sequences restrict transgene expression to hypertrophic cartilage in mice. Am. J. Pathol..

[238] Duncan S., Delage S., Chioran A., Sirbu O., Brown T.J., Ringuette M.J. (2020). The predicted collagen-binding domains of *Drosophila* SPARC are essential for survival and for collagen IV distribution and assembly into basement membranes. Dev. Biol..

[239] Leikina E., Mertts M.V., Kuznetsova N., Leikin S. (2002). Type I collagen is thermally unstable at body temperature. Proc. Natl. Acad. Sci. USA.

[240] Lin J., Zou B., Li H., Wang J., Li S., Cao J., Xie D., Wang F. (2024). Collagen XVII promotes dormancy of colorectal cancer cells by activating mTORC2 signaling. Cell. Signal..

[241] Ansorge H.L., Meng X., Zhang G., Veit G., Sun M., Klement J.F., Beason D.P., Soslowsky L.J., Koch M., Birk D.E. (2009). Type XIV collagen regulates fibrillogenesis. J. Biol. Chem..

[242] Berthod F., Germain L., Guignard R., Lethias C., Garrone R., Damour O., Rest M.V.D., Auger F.A. (1997). Differential expression of collagens XII and XIV in human skin and in reconstructed skin. J. Investig. Dermatol.

[243] Blaschke U.K., Eikenberry E.F., Hulmes D.J.S., Galla H.-J., Bruckner P. (2000). Collagen XI nucleates self-assembly and limits lateral growth of cartilage fibrils. J. Biol. Chem..

[244] Cancel M., Grimard G., Thuillard-Crisinel D., Moldovan F., Villemure I. (2009). Effects of in vivo statis compressive loading on aggrecan and type II and X collagens in the rat growth plate extracellular matrix. Bone.

[245] Dreier R., Opolka A., Grifka J., Bruckner P., Grassel S. (2008). Collagen IX-deficiency seriously compromises growth cartilage development in mice. Matrix Biol..

[246] Grassel S., Tan E.M.L., Timpl R., Chu M.-L. (1998). Collagen type XVI expression is modulated by basic fibroblast growth factor and transforming growth factor-β. FEBS Lett..

[247] Ruzzi L., Posteraro P., Zambruno G., Castiglia D., DAlessio M., Pas H., Mazzanti C., Didona B., Owaribe K., Meneguzzi G. (2001). A homozygous nonsence mutation in type XVII collagen gene (COL17A1) uncovers an alternatively spliced mRNA accounting for an unusually mild form of non-herlitz junctional epidermolysis bullosa. J. Investig. Dermatol..

[248] Kapyla J., Jaalinoja J., Tulla M., Ylostalo J., Nissinen L., Viitasalo T., Vehvilainen P., Marjomaki B., Nykvist P., Saamanen A.-M. (2004). The fibril-associated collagen IX provides a novel mechanism for cell adhesion to cartilaginous matrix. J. Biol. Chem..

[249] Liu X., Su J., Zhou H., Zeng Z., Li Z., Xiao Z., Zhao M. (2022). Collagen VI antibody reduces atherosclerosis by activating monocyte/macrophage polarization in *ApoE* mice. Int. Immunopharmacol..

[250] Soderhall C., Marenholz I., Kerscher T., Ruschendorf F., Esparza-Gordillo J., Worm M., Gruber C., Mayr G., Albrecht M., Rohde K. (2007). Variants in a novel epidermal collagen gene (COL29A1) are associated with atopic dermatitis. PLoS Biol..

[251] Kalchishkova N., Heinegard D., Blom A. (2009). The cartilage-specific collagen IX and its role in modulation of the complement activity. Mol. Immunol..

[252] Veit G., Zimina E.P., Franzke C.-W., Kutsch S., Siebolds U., Gordon M.K., Bruckner-Tuderman L., Koch M. (2007). Shedding of collagen XXIII is mediated by furin and depends on the plasma membrane microenvironment. J. Biol. Chem..

[253] Vijayasarathy M., Balaram P. (2019). Cone snail prolyl-4-hydroxylase a-subunit sequences derived from transcriptomic data and mass spectrometric analysis of variable proline hydroxylation in *C. amadis* venom. J. Proteom..

[254] Borges L., Logan M., Weber S., Lewis S., Fang C., Correr-Sobrinho L., Pfeifer C. (2024). Multi-acrylamides improve bond stability through collagen reinforcement under physiological conditions. Dent. Mater..

[255] Bos K.J., Rucklidge G.J., Dunbar B., Robins S.P. (1999). Primary structure of the helical domain of porcine collagen X. Matrix Biol..

[256] Kvist A.-P., Latvanlehto A., Sund M., Eklund L., Vaisanen T., Hagg P., Sormunen R., Komulainen J., Fassler R., Pihlajaniemi T. (2001). Lack of cytosolic and transmembrane domains of type XIII collagen results in progressive myopathy. Am. J. Pathol..

[257] Lai C.-H., Chu M.-L. (1996). Tissue distribution and developmental expression of type XVI collagen in the mouse. Tissue Cell.

[258] Mutolo M.J., Morris K.J., Leir S.-H., Caffrey T.C., Lewandowska M.A., Hollingsworth M.A., Harris A. (2012). Tumor suppression by collagen XV is independent of the restin domain. Matrix Biol..

[259] Myers J.C., Li D., Amenta P.S., Clark C.C., Nagaswami C., Weisel J.W. (2003). Type XIX collagen purified from human Umbilical cord if characterized by multiple sharp kinks delineating collagenous subdomains and by intermolecular aggregates via globular, disulfide-linked, and heparin-biding amino termini. J. Biol. Chem..

[260] Seppanen A., Suuronen T., Hofmann S.C., Majamaa K., Alafuzoff I. (2007). Distribution of collagen XVII in the human brain. Brain Res..

[261] Tuckwell D. (2002). Identification and analysis of collagen α1(XXI), a novel member of the FACIT collagen family. Matrix Biol..

[262] Zeltz C., Kusche-Gullberg M., Heljasvaara R., Gullberg D. (2023). Novel roles for cooperating collagen receptor families in fibrotic niches. Curr. Opin. Cell Biol..

[263] Zhang D., Zhu H., Harpaz N. (2016). Overexpression of a1 chain of type XI collagen (COL11A1) aids in the diagnosis of invasive carcinoma in endoscopically removed malignant colorectal polyps. Pathol. Res. Pract..

[264] Toumpoulis I.K., Oxford J.T., Cowan D.B., Anagnostopoulos C.E., Rokkas C.K., Chamogeorgakis T.P., Angouras D.C., Shemin R.J., Novab M., Ericsson M. (2009). Differential expression of collagen type V and XI a01 in human ascending thoracic aortic aneurysms. Ann. Thorac. Surg..

[265] Zwolanek D., Veit G., Eble J.A., Gullberg D., Ruggiero F., Heino J., Meier M., Stetefeld J., Koch M. (2014). Collagen XXII binds to collagen-binding integrins via the novel motifs GLQGER and GFKGER. Biochem. J..

[266] Yan W., Huang C., Yan Y., Wang P., Yuwen W., Zhu C., Fu R., Duan Z., Fan D. (2024). Expression, characterization and antivascular activity of amino acid sequence repeating collagen hexadecapeptide. Int. J. Biol. Macromol..

[267] Sanapalli B.K.R., Yele V., Singh M.K., Thumbooru S.N., Parvathaneni M., Karri V.V.S.R. (2023). Human beta defensin-2 loaded PLGA nanoparticles impregnated in collagen-chitosan composite scaffold for the management of diabetic wounds. Biomed. Pharmacother..

[268] Seppanen A., Autio-Harmainen H., Alafuzoff I., Sarkioja T., Veijola J., Hurskainen T., Brucker-Tuderman L., Tasanen K., Majamaa K. (2006). Collagen XVII is expressed in human CNS neurons. Matrix Biol..

[269] Jackow J., Schlosser A., Sormunen R., Nystrom A., Sitaru C., Tasanen K., Bruckner-Tuderman L., Franzke C.-W. (2016). Generation of a functional non-shedding collagen XVII mouse model: Relevance of collagen XVII shedding in wound healing. J. Investig. Dermatol..

[270] Ratzinger S., Eble J.A., Pasoldt A., Opolka A., Rogler G., Grifka J., Grassel S. (2010). Collagen XVI induces formation of focal contacts on intestinal myofibroblasts isolated from the normal and inflamed intestinal tract. Matrix Biol..

[271] Bonnet I., Cadau S., Berthelemy N., Bardey V., Andre-Frei V., Chavan M., Zahouani H., Rousselle P. (2017). 447 collagen XVIII, a key interfacial component of the skin architecture. J. Investig. Dermatol..

[272] Bracher S., Voumard B., Simon M., Kochetkova T., Pretterklieber M., Zysset P. (2024). Bone collagen tensile properties of the aging human proximal femur. Bone Rep..

[273] Brown J.C., Golbik R., Mann K., Timpl R. (1994). Structure and stability of the triple-helical domains of human collagen XIV. Matrix Biol..

[274] Akagi A., Tajima S., Ishibashi A., Matsubara Y., Takehana M., Kobayashi S., Yamaguchi N. (2002). Type XVI collagen is expressed in factor XIIIa^+^ monocyte-derived dermal dendrocytes and constitutes a potential substrate for factor XIIIa. J. Investig. Dermatol..

[275] Wirz J.A., Boudko S.P., Lerch T.F., Chapman M.S., Bachinger H.P. (2011). Crystal structure of the human collagen XV trimerization domain: A potent trimerizing unit common to multiplexin collagens. Matrix Biol..

[276] Kvist A.-P., Latvanlehto A., Sund M., Horelli-Kuitunen N., Rehn M., Palotie A., Beier D., Pihlajaniemi T. (1999). Complete exon-intron organization and chromosomal location of the gene for mouse type XIII collagen (*col13a1*) and comparison with its human homologue. Matrix Biol..

[277] Wu Y., Sun N.-N., Dang E.-L., Jin L., Liu Z.-F., Zhang W., Yang L.-T., Wang G. (2013). Anti-collagen XVII single-chain Fv antibody blocks the autoimmune reaction mediated by pathogenic autoantibodies in bullous pemphigoid. J. Dermatol. Sci..

[278] Smolen J.S., Aletaha D., McInnes I.B. (2016). Rheumatoid arthritis. Lancent.

[279] Yan L., Zhang Y., Zhang Y., Chen Q., Zhang L., Han X., Yang Y., Zhang C., Liu Y., Yu R. (2024). Preparation and characterization of a novel humanized collagen III with repeated fragments of Gly300-Asp329. Protein Expr. Purif..

[280] Tian P., Koudis N.-M., Morais M.R.P.T., Pickard A., Fresquet M., Adamson A., Derby B., Lennon R. (2024). Collagen IV assembly is influenced by fluid flow in kidney cell-derived matrices. Cells Dev..

[281] Hudsom D.M., Weis M.A., Jeong K.S., Dimori M., Lee B.H., Morello R., Eyre D.R. (2017). P3h3-null and Sc65-null mice phenocopy the collagen lysine under-hydroxylation and cross-linking abnormality of Ehlers-Danlos syndrome type VIA. J. Biol. Chem..

[282] Dilley K.K., Prasad K.R., Nguyen T.V., Stokolosa A., Borden P.A., Heur J.M., Kim S., Hill M.G., Wong B.J.F. (2024). Second harmonic generation microscopy of electromechanical reshaping on corneal collagen. Exp. Eye Res..

[283] Exposito J.-Y., Larroux C., Cluzel C., Valcourt U., Lethias C., Degnan B.M. (2008). Demosponge and sea anemone fibrillar collagen diversity reveals the early emergence of A/C clades and the maintenance of the modular structure of type V/XI collagens from sponge to human. J. Biol. Chem..

[284] Watanabe M., Natsuga K., Nishie W., Donati G., Fujimura Y., Tsukiyama T., Ujiie h., Ozaki M., Watt F.M., Shimizu H. (2017). 085 type XVII collagen suppresses interfollicular epidermal proliferation in neonatal and aged skin, and helps rejuvenate epidermis. J. Investig. Dermatol..

[285] Wilhelm D., Wurtz A., Abouelfarah H., Sanchez G., Bui C., Vincourt J.-B. (2023). Tissue-specific collagen hydroxylation at GEP/GDP triplets mediated by P4HA2. Matrix Biol..

[286] Schonborn K., Willenborg S., Schulz J.-N., Imhof T., Eming S.A., Quondamatteo F., Brinckmann J., Niehoff A., Paulsson M., Koch M. (2020). Role of collagen XII in skin homeostasis and repair. Matrix Biol..

[287] Rempfer C., Hoernstein S.N.W., Gessel N.V., Graf A.W., Spiegelhalder R.P., Bertolini A., Bohlender L.L., Parsons J., Decker E.L., Reski R. (2024). Differential prolyl hydroxylation by six physcomitrella prolyl-4 hydroxylases. Comput. Struct. Biotechnol. J..

[288] Yeh S.-I., Han K.-Y., Sabri A., Rosenblatt M.I., Azar D.T., Jain S., Chang J.-H. (2010). MMP-7 knock-in corneal fibroblast cell lines secrete MMP-7 with proteolytic activity towards collagen XVIII. Curr. Eye Res..

[289] Zhang L., Zhang S., Song H., Li B. (2018). Effect of collagen hydrolysates from silver carp skin (*Hypophthalmichthys molitrix*) on osteoporosis in chronologically aged mice: Increasing bone remodeling. Nutrient.

[290] Zhang H., Zhao Y., Hou D. (2024). The research of collagen for tissue repair in China from 2019 to 2023 based on bibliometrics and visualization analysis. Med. Novel. Technol. Devices.

[291] Tompson S.W., Bacino C.A., Safina N.P., Bober M.B., Proud V.K., Funari T., Wangler M.F., Nevarez L., Ala-kokko L., Wilcox W.R. (2010). Fibrochondrogenesis results from mutations in the *COL11A1* type XI collagen gene. AJHG.

[292] Wang Q., Yan H., Yao L., Xie Y., Liu P., Xiao J. (2024). A highly bioactive THPC-crosslinked recombinant collagen hydrogel implant for aging skin rejuvenation. Int. J. Biol. Macromol..

[293] Rinta-Jaskari M.M., Naillat F., Ruotsalainen H.J., Koivunen J.T., Sasaki T., Pietila I., Elamaa H.P., Kaur I., Manninen A., Vainio S.J. (2023). Temporally and spatially regulated collagen XVIII isoforms are involved in ureteric tree development via the TSP1-like domain. Matrix Biol..

[294] Fertala J., Arita M., Steplewski A., Arnold W.V., Fertala A. (2018). Epiphyseal growth plate architecture is unaffected by early postnatal activation of the expression of R992C collagen II mutant. Bone.

[295] Fitzgerald J., Holden P., Hansen U. (2013). The expanded collagen VI family: New chains and new questions. Connect. Tissue Res..

[296] Deymier A.C., Deymier P.A. (2024). Open-system force-elongation relationship of collagen in chemo-mechanical equilibrium with water. J. Mechanical. Behav. Biomed. Mater..

[297] Foley J.E., Koch M., Gerecke D.R., Fitch J.M., Gordon M.K. (1997). An NC1 domain splicing variant of type XII collagen isolated from a corneal cDNA library. Matrix Biol..

[298] Steplewski A., Brittingham R., Jimenez S.A., Fertala A. (2005). Single amino acid substitutions in the C-terminus of collagen II alter its affinity for collagen IX. Biochem. Biophys. Res. Commun..

[299] Sumiyoshi H., Inoguchi K., Khaleduzzaman M., Ninomiya Y., Yoshiokda H. (1997). Ubiquitous expression of the α1(XIX) collagen gene (*Col19α1*) during mouse embryogenesis becomes restricted to a few tissues in the adult organism. J. Biol. Chem..

[300] Bretaud S., Guillon E., Karppinen S.-M., Pihlajaniemi T., Ruggiero F. (2020). Collagen XV, a multifaceted multiplexin present across tissues and species. Matirx Biol. Plus.

[301] Selvaraj V., Sekaran S., Dhanasekaran A., Warrier S. (2024). Type 1 collagen: Synthesis, structure and key functions in bone mineralization. Differentiation.

[302] Kinnunen A.I., Sormunen R., Elamaa H., Seppinen L., Miller R.T., Ninomiya Y., Janmey P.A., Pihlajaniemi T. (2011). Lack of collagen XVIII long isoforms affects kidney podocytes, whereas the short form is needed in the proximal tubular basement membrane. J. Biol. Chem..

[303] Krasue M.B., Vang N., Ahlmeyer L., Tung T.A. (2023). Effects of lipid extraction on human bone collagen: Comparing stable carbon and nitrogen isotope values with the without lipid extraction. J. Archaeol. Sci. Rep..

[304] Palamae S., Patil U., Suyapoh W., Sornying P., Buatong J., Zhang B., Benjakul S. (2024). Elucidation of high-pressure processing toward microbial inhibition, physicochemical properties, collagen fiber and muscle structure of blood calm edible portion. Food Chem..

[305] Skov K., Oxfeldt M., Thogersen R., Hansen M., Bertram H.C. (2019). Enzymatic hydrolysis of a collagen hydrolysate enhances postprandial absorption rate- A randomized controlled trial. Nutrients.

[306] Smith S.M., Zhang G., Birk D.E. (2014). Collagen V localizes to pericellular sites during tendon collagen fibrillogenesis. Matirx Biol..

[307] Warner L.R., Brown R.J., Yingst S.M.C., Oxford J.T. (2006). Isoform-specific heparan sulfate binding withing the amino-terminal noncollagenous domain collagen α1(XI). J. Biol. Chem..

[308] Rinta-Jaskari M.M., Naillat F., Ruotsalainen H.J., Ronkainen V.-P., Heljaasvaara R., Akram S.U., Izzi V., Miinalainen I., Vainio S.J., Pihlajaniemi T.A. (2024). Collagen XVIII regulates extracellular matrix integrity in the developing nephrons and impacts nephron progenitor cell behavior. Matrix Biol..

[309] Parikka M., Nissinen L., Kainulainen T., Bruckner-Tuderman L., Salo T., Heino J., Tasanen K. (2006). Collagen XVII promotes integrin-mediated squamous cell carcinoma transmigration-A novel role for aII_b_ integrin and tirofiban. Exp. Cell Res..

[310] Parsons P., Gilbert S.J., Vaughan-Thomas A., Sorrell D.A., Notman R., Bishop M., Hayes A.J., Mason D.J., Duance V.C. (2011). Type IX collagen interacts with fibronectin providing an important molecular bridge in articular cartilage. J. Biol. Chem..

[311] Paul C., Leser S., Oesser S. (2019). Significant amounts of functional collagen peptides can be incorporated in the diet while maintaining indispensable amino acid balance. Nutrients.

[312] Ma L., Liang X., Yu S., Zhou J. (2022). Expression, characterization, and application potentiality evaluation of recombinant human-like collagen in *Pichia pastoris*. Bioresour. Bioprocess.

[313] Manferdini C., Zini N., Gabusi E., Paolella F., Lambertini E., Penolazzi L., Piva R., Lisignoli G. (2018). Immunoelectron microscopis localization of collagen type XV during human mesenchymal stem cells mineralization. Connect. Tissue Res..

[314] Kassner A., Tiedemann K., Botbohm H., Ludwig T., Morgelin M., Reinhardt D.P., Chu M.-L., Bruckner P., Grassel S. (2004). Molecular structure and interaction of recombinant human type XVI collagen. J. Mol. Biol..

[315] Katayama K., Kuriki M., Kamiya T., Tochigi Y., Suzuki H. (2018). Giantin is required for coordinated production of aggrecan, link protein and type XI collagen during chondrogenesis. Biochem. Biophys. Res. Commun..

[316] Kaur H., Singh M., Kaur N., Pati P.K., Rani M., Kang T.S. (2024). Sustainable dissolution of collagen and the formation of polypeptides in deep eutectic solvents for application as antibacterial agents. RSC Sustain..

[317] Chen Y., Sumiyoshi H., Oxford J.T., Yoshioka H., Ramirez F., Morris N.P. (2001). *Cis*-acting elements regulate alternative splicing of exons 6A, 6B, and 8 of the a1(XI) collagen gene and contribute to the regional diversification of collagen XI matrices. Matrix. Biol..

[318] Chen Y., Forster L., Wang K., Gupta H.S., Li X., Huang J., Rui Y. (2024). Investigation of collagen reconstruction mechanism in skin wound through dual-beam laser welding: Insights from multi-spectroscopy, molecular dynamics simulation, and finite element Multiphysics simulation. J. Photochem. Photobiol. Biol..

[319] Chiquet M., Birk D.E., Bonnemann C.G., Koch M. (2014). Collagen XII: Protecting bone and muscle integrity by organizing collagen fibrils. Int. J. Biochem. Cell Biol..

[320] Lui V.C.H., Ng L.J., Sat E.W.Y., Cheah K.S.E. (1996). The human a2(XI) collagen gene (COL11A2): Completion of coding information, identification of the promoter sequence, and precise localization within the major histocompatibility complex reveal overlap with the KE5 gene. Genomics.

[321] Kroeger J., Hofmann S., Leppert J., Has C., Franzke C. (2017). 098 novel amino acid duplication in collagen XVII causes mild junctional epidermolysis bullosa by altering the coiled-coil structure and impairing collagen XVII trimerization and maturation. J. Investig. Dermatol..

[322] Stephan S., Sherratt M.J., Hodson N., Shuttleworth C.A., Kielty C.M. (2004). Expression and supramolecular assembly of recombinant a1(VIII) and a2(VIII) collagen homotrimers. J. Biol. Chem..

[323] Kroeger J., Hoppe E., Galiger C., Has C., Franzke C.-W. (2019). Amino acid substitution in the C-terminal domain of collagen XVII reduces laminin-332 interaction causing mild skin fragility with atrophic scarring. Matrix. Biol..

[324] Wang Q., Yan H., Yao L., Li W., Xiao J. (2024). A highly durable and biocompatible bionic collagen implant with exceptional anti-calcification and collagen regeneration capabilities for improved skin rejuvenation. Mater. Des..

[325] Sadri G., Fischer A.G., Brittian K.R., Elliott E., Nystoriak M.A., Uchida S., Wysoczynski M., Leask A., Jones S.P., Moore J.B. (2022). Collagen type XIX regulates cardiac extracellular matrix structure and ventricular function. Matrix Biol..

[326] Moreno-Ricardo M.A., Gomez-Contreras P., Gonzalez-Delgado A.D., Hernandez-Fernandez J., Ortega-Toro R. (2024). Development of films based on chitosan, gelatin and collagen extracted from bocachico scales (*Prochilodus magdalenae*). Heliyon.

[327] Mortensen J.H., Olesen M.L., Jensen C., Willumsen N., Giuffrida P., Pinzani M., Mazza G., Sabatino A.D., Karsdal M., Manon-Jensen T. (2018). Sa 1826- Serological assessment of type XVI collagen reflects intestinal structures in Crohns disease patients. Gastroenterology.

[328] Munezane H., Oizumi H., Wakabayashi T., Nishio S., Hirasawa T., Sato T., Harada A., Yoshida T., Eguchi T., Yamanashi Y. (2019). Roles of collagen XXV and its putative receptors PTPϭ/δ in intramuscular motor innervation and congenital cranial dysinnervation disorder. Cell Rep..

[329] Ockleford C.D., McCracken S.A., Rimmington L.A., Hubbard A.R.D., Bright N.A., Cockcroft N., Jefferson T.B., Waldron E., DLacey C. (2013). Type VII collagen associated with the basement membrane of amniotic epithelium forms giant anchoring rivets which penetrate a massive lamina reticularis. Placenta.

[330] Lunstrum G.P., McDonough A.M., Marinkovich M.P., Keene D.R., Morris N.P., Burgeson R.E. (1992). Identification and partial purification of a large, variant form of type XII collagen. J. Biol. Chem..

[331] Stape T.H.S., Mutluay M.M., Tjäderhane L., Uurasjärvi E., Koistinen A., Tezvergil-Mutluay A. (2021). The pursuit of resin-dentin bond durability: Simultaneous enhancement of collagen structure and polymer network formation in hybrid layers. Dent. Mater..

[332] Hamill K.J., Hopkinson S.B., Jonkman M.F., Jones J.C.R. (2011). Type XVII collagen regulates lamellipod stability, cell motility, and signaling to Rac1 by targeting bullous pemphigoid antigen 1e to α6β4 integrin. J. Biol. Chem..

[333] Hecht J.T., Makitie O., Hayes E., Haynes R., Susic M., Montufar-Solis D., Duke P.J., Cole W.G. (2004). Chondrocyte cell death and intracellular distribution of COMP and type IX collagen in the pseudoachondroplasia growth plate. J. Orthop. Res..

[334] Hennet T. (2019). Collagen glycosylation. Curr. Opin. Struct. Biol..

[335] Hirano S., Yonezawa T., Hasegawa H., Hattori S., Greenhill N.S., Davis P.F., Sage E.H., Ninomiya Y. (2004). Astrocytes express type VIII collagen during the repair process of brain cold injury. Biochem. Biophys. Res. Commun..

[336] Hjorten R., Hansen U., Underwood R.A., Telfer H.E., Fernandes R.J., Krakow D., Sebald E., Wachsmann-Hogiu S., Bruckner P., Jacquet R. (2007). Type XXVII collagen at the transition of cartilage to bone during skeletogensis. Bone.

[337] Huang Y., Deng H., Zhang J., Sun H., Li W., Li C., Zhang Y., Sun D. (2021). A photoelectrochemical immunosensor based on ReS_2_ nanosheets for determination of collagen III related to abdominal aortic aneurysm. Microchem. J..

[338] Danfelter M., Onnerfjord P., Heinegard D. (2007). Fragmentation of proteins in cartilage treated with interleukin-1: Specific cleavage of type IX collagen by matrix metalloproteinase 13 releases the NC4 domain. J. Biol. Chem..

[339] Brull A., Sarathy A., Bolduc V., Chen G.S., McCarty R.M., Bonnemann C.G. (2024). Optimized allele-specific silencing of the dominant-negative *COL6A1 G293R* substitution causing collagen VI-related dystrophy. Mol. Ther. Nucleic Acids.

[340] Fukuta S., Oyama M., Kavalkovich K., Fu F.H., Niyibizi C. (1998). Identification of types II, IX, and X collagens at the insertion site of the bovine achilles tendon. Matrix Biol..

[341] Tajima S., Akagi A., Tanaka N., Ishibashi A., Kawada A., Yamaguchi N. (2000). Expression of type XVI collagen in cultured skin fibroblasts is related to cell growth arrest. FEBS Lett..

[342] Tao G., Levay A.K., Peacock J.D., Huk D.J., Both S.N., Purcell N.H., Pinto J.R., Galantowicz M.L., Koch M., Lucchesi P.A. (2012). Collagen XIV is important for growth and structural integrity of the myocardium. J. Mol. Cell. Cardiol..

[343] Hamada Y., Sumiyoshi H., Matsuo N., Yun-Feng W., Nakashima M., Yanagisawa S., Yoshioka H. (2010). The pro-a2(XI) collagen gene is expressed in odontoblasts. Biochem. Biophys. Res. Commun..

[344] Suzuki N., Asamura K., Kikuchi Y., Takumi Y., Abe S., Imamura Y., Hayashi T., Aszodi A., Fassler R., Usami S.-I. (2005). Type IX collagen knock-out mouse shows progressive hearing loss. Neurosci. Res..

[345] Gebauer J.M., Kobbe B., Paulsson M., Wagener R. (2016). Structure, evolution and expression of collagen XXVIII: Lessons from the zebrafish. Matrix Biol..

[346] Giacomazza D., Viappiani C., Cera E.D., Musio C. (2017). SIBPA under the Tuscan sun: Introduction to the SIBPA XXIII special issue. Biophys. Chem..

[347] Gibney R., Patterson J., Ferraris E. (2021). High-resolution bioprinting of recombinant human collagen type III. Polymers.

[348] Seppinen L., Sormunen R., Soini Y., Elamaa H., Heljasvaara R., Pihlajaniemi T. (2008). Lack of collagen XVIII accelerates cutaneous wound healing, while overexpression of its endostatin domain leads to delayed healing. Matrix Bio..

[349] Inoue O., Suzuki-Inoue K., Ozaki Y. (2008). Redundant mechanism of platelet adhesion to laminin and collagen under flow: Involvement of von Willebrand factor and glycoprotein IB-IX-V. J. Biol. Chem..

[350] Ito K., Maeda K., Kariya M., Yasui K., Araki A., Takahashi Y., Takakura Y. (2023). Formation of DNA nanotubes increases uptake into fibroblasts via enhanced affinity for collagen. Int. J. Pharm..

[351] Llacua L.A., Hoek A., Haan B.J.D., Vos P.D. (2018). Collagen type VI interaction improves human islet survival in immunoisolating microcapsules for treatment of diabetes. Islets.

[352] Cao L., Zhang Z., Yuan D., Yu M., Min J. (2024). Tissue engineering applications of recombinant human collagen: A review of recent progress. Front. Bioeng. Biotechnol..

[353] South A.P., Laimer M., Gueye M., Sui J.Y., Eichenfield L.F., Mellerio J.E., Nystrom A. (2023). Type VII collagen deficiency in the oncogenesis of cutaneous squamous cell carcinoma in dystrophic epidermolysis bullosa. J. Investig. Dermatol..

[354] Davis P.F., Ryan P.A., Kittelberger R., Greenhill N.S. (1990). The collagenous protein with elastin crosslinks from Descemet’s membrane is not related to type VIII collagen. Biochem. Biophys. Res. Commun..

[355] Izu Y., Adams S.M., Connizzo B.K., Beason D.P., Soslowsky L.J., Koch M., Birk D.E. (2021). Collagen XII mediates cellular and extracellular mechanisms regulate establishment of tendon structure and function. Matrix Biol..

[356] Huang H., Song X., Zhang J., Fan Y., Kong M., Zhang L., Hou H. (2024). Novel collagen gradient membranes with multiphasic structures: Preparation, characterization, and biocompatibility. Colloids Surf. Biointerfaces.

[357] Garcia-Berbel P., Cadenas N., Azueta A., Freire J., Gonzalez-Sanchez R., Pereda S., Portillo J., Gomez-Roman J. (2014). Collagen, type xi, alpha 1 is expressed in neoplastic cells with sarcomatoid transformation in renal cell carcinoma. Pathology.

[358] Oertzen-Hagemann V., Kirmse M., Eggers B., Pfeiffer K., Marcus K., Marees M.D., Platen P. (2019). Muscle proteome in recreationally active men. Nutrients.

[359] Pihlajaniemi T., Rehn M. (1995). Two new collagen subgroups: Membrane-associated collagens and types XV and XVIII. Progress Nucleic Acid Res. Mol. Biol..

[360] Savage B., Ginsberg M.H., Ruggeri Z.M. (1999). Influence of fibrillar collagen structure on the mechanisms of platelet thrombus formation under flow. Blood.

[361] Sanderg-Lall M., Hagg P.O., Wahlstrom I., Pihlajaniemi T. (2000). Type XIII collagen is widely expressed in the adult and developing human eye and accentuated in the ciliary muscle, the optic nerve and the neural retina. Exp. Eye. Res..

[362] Sasaki T., Larsson H., Tisi D., Claesson-Welsh L., Hohenester E., Timpl R. (2000). Endostatins derived from collagens XV and XVIII differ in structural and binding properties, tissue distribution and anti-angiogenic activity. J. Mol. Biol..

[363] Sun W., Shahrajabian M.H., Wang N. (2025). A study of the different strains of the genus *Azospirillum* spp. on increasing productivity and stress resilience in plants. Plants.

[364] Sun W., Shahrajabian M.H. (2025). Biostimulant and beyong: *Bacillus* spp., the important plant growth-promoting rhizobacteria (PGPR)-based biostimulant for sustainable agriculture. Earth Syst. Environ..

